# Evaluation of the performance of yielding rockbolts during rockbursts using numerical modeling method

**DOI:** 10.1007/s40789-022-00537-6

**Published:** 2022-11-22

**Authors:** Jun Wang, Derek B. Apel, Huawei Xu, Chong Wei

**Affiliations:** School of Mining and Petroleum Engineering, University of Alberta, T6G 2R3 Edmonton, AB Canada

**Keywords:** Rockburst, Rockburst damage, Yielding rockbolt, Numerical modeling, UDEC, Underground mining

## Abstract

The assessment of yielding rockbolt performance during rockbursts with actual seismic loading is essential for rockburst supporting designs. In this paper, two types of yielding rockbolts (D-bolt and Roofex) and the fully resin-grouted rebar bolt are modeled via the “rockbolt” element in universal distinct element code (UDEC) after an exact calibration procedure. A two-dimensional (2D) model of a deep tunnel is built to fully evaluate the performance (e.g., capacity of energy-absorption and control of rock damage) of yielding and traditional rockbolts based on the simulated rockbursts. The influence of different rockburst magnitudes is also studied. The results suggest that the D-bolt can effectively control and mitigate rockburst damage during a weak rockburst because of its high strength and deformation capacity. The Roofex is too “soft” or “smooth” to limit the movement of ejected rocks and restrain the large deformation, although it has an excellent deformation capacity. The resin-grouted rebar bolt can maintain a high axial force level during rockbursts but is easy to break during dynamic shocks, which fails to control rapid rock bulking or ejection. Three types of rockbolts cannot control the large deformation and mitigate rockburst damage effectively during violent rockbursts. The rockburst damage severity can be significantly reduced by additional support with cable bolts. This study highlights the effectiveness of numerical modeling methods in assessing the complex performance of yielding rockbolts during rockbursts, which can provide some references to improve and optimize the design of rock supporting in burst-prone grounds.

## Highlights

(1) Energy-absorption capacity of yielding rockbolts in rockbursts is evaluated.

(2) Rock deformation and rockburst damage are quantitatively assessed.

(3) Performance of yielding rockbolts in weak and strong rockbursts is studied.

(4) Effects of floor support and cable bolts on controlling rockbursts are discussed.

**List of symbols**

**Table:** 

2D	Two-dimensional	DEM	Distinct element method
PPV	Peak particle velocity	FLAC3D	Fast Lagrangian analysis of continua in 3 dimensions
*m*_i_	Material constant for intact rocks	Δ*F*_*t*_	Incremental axial force in a “rockbolt” element
*m*_b_	Material constant for rock masses	*E*	Young’s modulus of the rockbolt
*s*	Material constant for rock masses	*A*	Cross-sectional area
*a*	Material constant for rock masses	*L*	Length of a “rockbolt” element
*ρ*	Bulk density of intact rocks	*F*_s_	Shear force that develops in the shear coupling spring
*σ*_ci_.	Uniaxial compressive strength (UCS) of intact rocks	*cs*_sstiff_	Coupling spring shear stiffness
*E*_i_	Young’s modulus of intact rocks	*u*_p_	Axial displacement of the rockbolt
*v*	Poisson’s ratio of intact rocks	*u*_m_	Axial displacement of the medium
*σ*_cm_	UCS of rock masses	*F*_s_^max^	Maximum shear force per length of the rockbolt
*E*_m_	Deformation modulus of rock masses	*cs*_scoh_	Cohesive strength of the shear coupling spring
*D*	Factor that depends upon the degree of disturbance	*σ’*_c_	Average effective confining stress perpendicular to the “rockbolt” element
GSI	Geological Strength Index	*cs*_sfric_	Friction angle of the shear coupling spring
ISRM	International Society for Rock Mechanics	*cs*_nstiff_	Coupling spring normal stiffness
*K*	Bulk modulus	*F*_n_	Normal force that develops in the normal coupling spring
*G*	Shear modulus	*u*_p_^*n*^	Displacement of the rockbolt perpendicular to the axial direction of the rockbolt
*k*_n_	Normal stiffness of contacts	*u*_m_^*n*^	Displacement of the medium (soil or rock) perpendicular to the axial direction of the rockbolt
*k*_s_	Shear stiffness of contacts	*F*_n_^max^	Maximum normal force per length of the rockbolt:
*c* ^*j*^	Cohesion force of contacts	*cs*_ncoh_	Cohesive strength of the normal coupling spring
*φ* ^*j*^	Friction angle of contacts	*cs*_nfric_	Friction angle of the normal coupling spring
*σ*_t_^*j*^	Tensile strength of contacts	*D*_c_	Total damage
Δ*σ*_n_	Normal stress increment	*D*_t_	Tensile damage
Δ*u*_n_	Normal displacement increment	*D*_s_	Shear damage
*σ*_n_	Normal stress	*L*_c_	Total length of failed contacts
UDEC	Universal distinct element code	*L*_t_	Length of failed contacts in tensile failure
*τ*_s_	Shear stress	*L*_s_	Length of failed contacts in shear failure
Δ*u*_s_^e^	Elastic shear displacement increment	M_L_	Richter magnitude
DDA	Discontinuous deformation analysis		

## Introduction

**Fig. 1:**
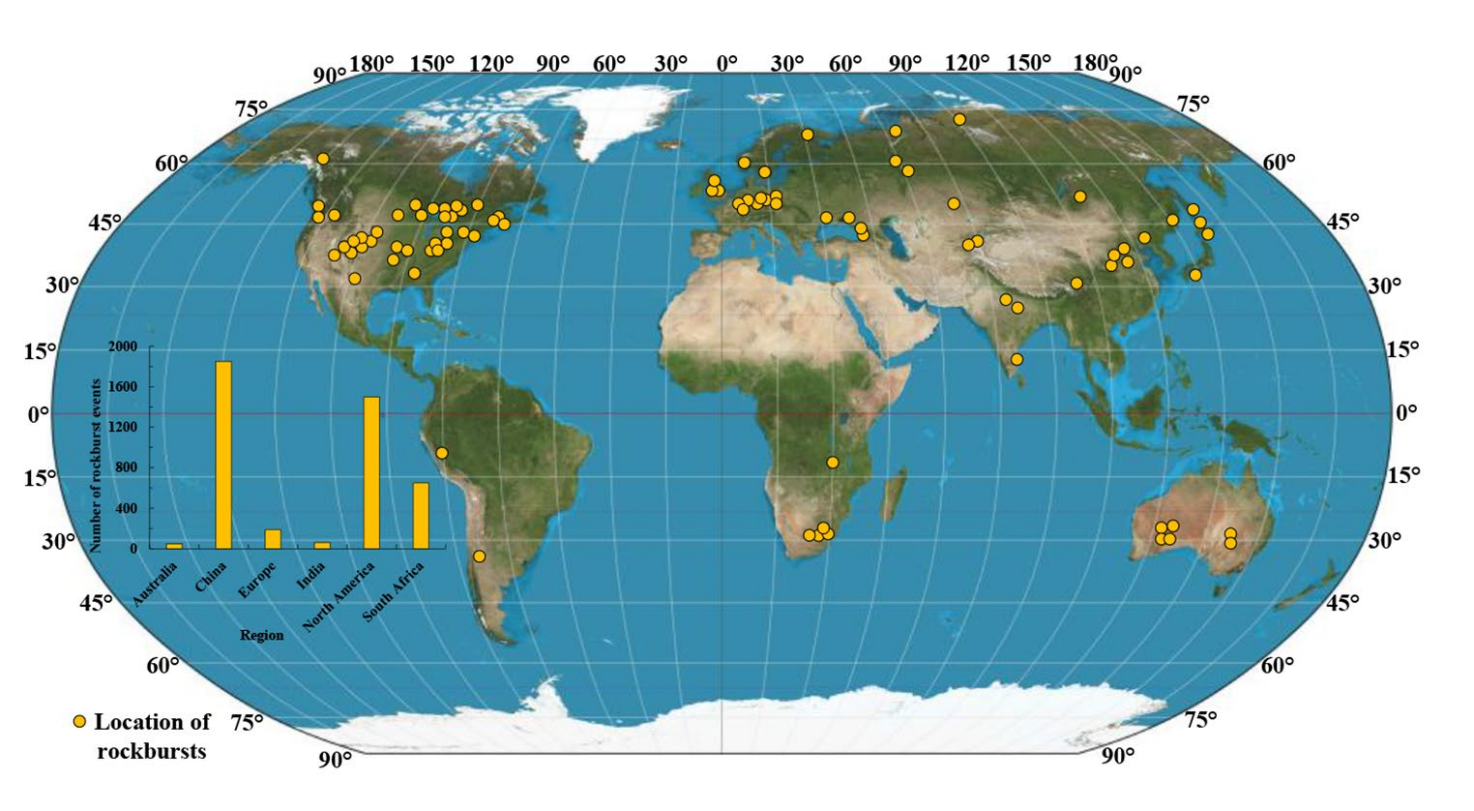
A historical rockburst map for 1931–2019 (updated after Bennett and Marshall 2001) The statistical data are incomplete

Rockburst is a seismic event characterized by the sudden ejection of rock materials from their surroundings, accompanied by a violent release of energy (Blake 1972; Cai 2013). The suddenly ejected rocks can damage equipment, facilities, and even cause fatalities (Naji et al. 2018; Pu et al. 2019; Zhang et al. 2012). The unpredictability and high destructiveness of rockburst have made it become one of the most hazardous geological disasters (Wang et al. 2021a, b). To date, rockburst events have been reported in all mining countries (e.g., South Africa, Canada, USA, Australia, Poland, China, Russia, India, and Chile) since the beginning of the 20th century (Blake and Hedley 2003). Many civil engineering projects such as deep tunnels in Switzerland, Norway, Iran, Peru, and China have also suffered rockburst problems (Dammyr 2016; Farrokh and Rostami 2009; Kaiser and Cai 2012; Zhang et al. 2012). Figure 1 shows a historical rockburst map of more than 4000 events from near 50 areas in the world in recent 90 years. The severity of those incidents and the large number of rockburst events are undisputed evidence that rockburst is a severe and global problem. This urges much work to be done to prevent and mitigate rockburst damage.

**Fig. 2:**
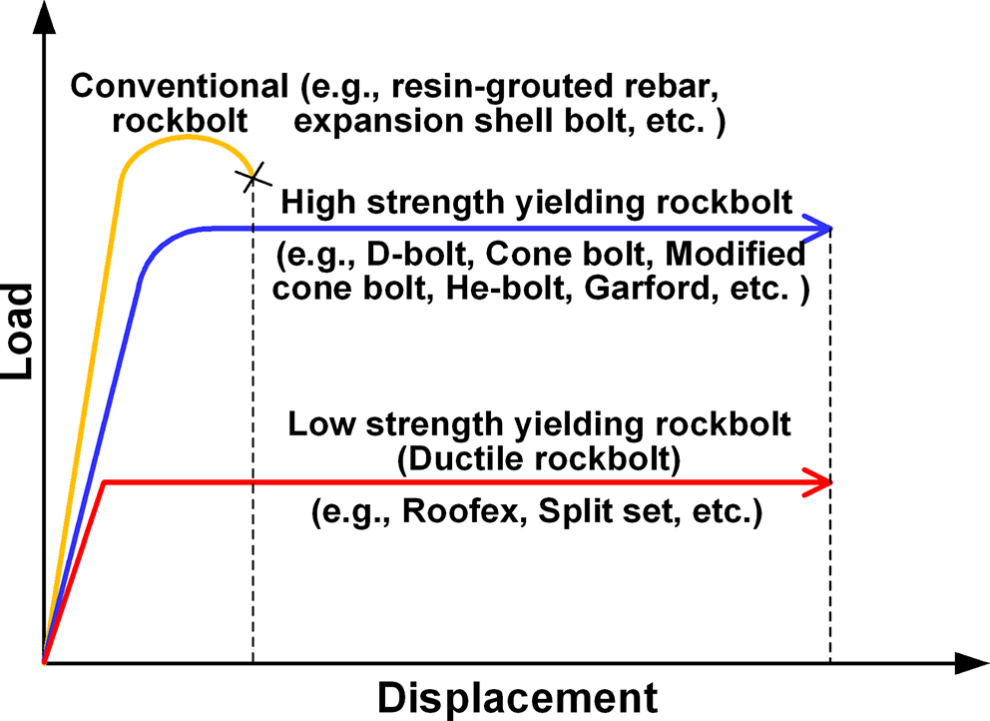
Load-displacement characteristics of conventional and yielding rockbolts (after Li et al. 2014)

It is always the best choice to prevent rockburst occurrence rather than to control and mitigate rockburst damage afterward. Methods that can avoid rockburst occurrence include choosing the rational location, size, and shape of openings, changing excavation methods and sequences, and using ground conditioning methods (e.g., distress drilling and blasting) (Cai 2013; Kaiser and Cai 2012; Mitri 2000). However, despite prevention measures being applied successfully in many cases, rockbursts can still occur due to the lack of sufficient geotechnical data and the complexity of rockburst mechanisms (Cai 2013; Ghorbani et al. 2020). Hence, the rock support system is usually required as the last defense line to control and mitigate rockburst damage. The rockburst support elements (e.g., rockbolts) in burst-prone grounds need to resist dynamic loads and accommodate large deformations caused by rock fracturing, dilation, and ejection (Kaiser and Cai 2012). Kaiser et al. 1996 reported that, generally, ejection velocities below 1.5 m/s could be handled by standard ground supports, but additional supports were required for higher velocities. Thus, support elements should allow yielding to absorb more kinetic energy and have higher displacement capacities than conventional support elements. This demand promotes the emergence and development of yielding or energy-absorption rockbolts. Figure 2 shows the typical load-displacement characteristics of yielding and conventional rockbolts.

As shown in Fig. 2, various types of yielding rockbolts have been invented due to the tireless efforts of researchers and engineers (Li et al. 2014; Sharifzadeh et al. 2020). Although the energy absorption mechanism (shank stretching, e.g., D-bolt; ploughing of anchors, e.g., cone bolt; extrusion of shanks, e.g., Roofex) might be different, some types of yielding rockbolts, e.g., Cone bolt (Ortlepp 1992), Roofex (Atlas Copco Construction Mining Technique 2009; Charette and Plouffe 2007), Garford bolt (Varden et al. 2008), D-Bolt (Li 2011; Normet 2021), Yield-Lok (Wu et al. 2011), and He-bolt (He et al. 2012) have been shown that they can control rockbursts effectively.

A critical task is to evaluate the performance (e.g., deformation, strength, control of rock damage and capacity of energy-absorption) of rockbolts before being widely used. The methodologies to study rockbolt behavior mainly include: field test (Kang et al. 2009; Li 2011; Wu et al. 2019), laboratory test, and numerical modeling. Field test method can obtain real-time data and assess the in situ performance of rockbolts, but they are usually time-consuming, expensive, and dangerous especially in burst-prone grounds. Compared with field tests, the experimental methods have the advantages of repeatability, safety, and flexibility (Zhang and Nordlund 2019). At present, significant efforts have been made to evaluate the static and dynamic performances of conventional and yielding rockbolts via laboratory tests, mainly including pull-out, shear, and drop tests (Cai et al. 2010; Charette and Plouffe 2007; Li 2011; Li and Doucet 2011; Skrzypkowski 2021; Skrzypkowski et al. 2020; Stillborg 1994; Stjern 1995). The research has achieved many positive outcomes, providing excellent references for understanding rockbolt behavior under different conditions. Nevertheless, it should be noted that experimental methods employ many idealized assumptions which are incapable of matching the actual field circumstances (Manouchehrian 2016). For instance, Bosman et al. (2018) stated that the dynamic capacity of a rockbolt is not a constant value, and the loading mode of a rockbolt will affect its dynamic capacity. The impact loading from conventional drop tests might not be representative of rockburst loading. Wu et al. (2019) also pointed out that the impact load in drop tests cannot represent the impact of ground pressure load, and the existing test system generally cannot reproduce the complex ground support/rock mass interaction that exists in an underground environment. Besides, original rock stress is not considered in tests. Therefore, the performance of yielding rockbolts during rockbursts with actual seismic loading is worth evaluating.

With the rapid development of information technology (IT) and computer equipment, various numerical methods and codes have been developed and employed to simulate complex physical phenomena in rock mechanics and rock engineering (Wang et al. 2021a). The numerical simulation methods have been acknowledged as an effective research and engineering design tools as it can represent the realistic mechanical behavior of rock masses and support elements with rational input data (e.g., excavation size and shape, material properties, and boundary conditions) and calibration procedures (Manouchehrian 2016). Hence, the actual engineering problems can be simulated and analyzed in detail and depth. Mortazavi and Tabatabaei Alavi (2013) employed fast Lagrangian analysis of continua in 3 dimensions (FLAC3D) software to study the behavior of fully grouted rebar rockbolts (with and without head plate) and the yielding rockbolt under dynamic loading. They concluded that the yielding rockbolt had the best performance in absorbing dynamic stress waves and controlling rock movement. Nie et al. (2014) developed rockbolt models using discontinuous deformation analysis (DDA) to investigate the failure mechanism of an expansion-shell bolt, fully grouted rebar, split set, and D-bolt in simulated pull-out and drop tests. Marambio et al. (2018) modeled a laboratory-scale test via FLAC3D to study the performance of threadbar in dynamic loading. The simulation results matched well with laboratory observations. Yokota et al. (2019) assessed a self-developed deformation-controlled rockbolt (DC-bolt)’s behavior in tunnel supporting via DDA simulation. Zhang and Nordlund (2019) employed universal distinct element code (UDEC) to investigate the differences of dynamic performances of a fully grouted rebar between the simulated drop tests and seismic loading in the configuration where two slightly separated rock bars were used. Zhao et al. (2021) studied the influence of structure element position on the anchoring effect of energy-absorption bolts via simulating pull-out tests.

In summary, most current work focuses on the evaluation of the effects of traditional rockbolts under dynamic loading while some researchers try to simulate the dynamic behavior of yielding rockbolts by reproducing drop tests. However, to the authors’ knowledge, few numerical studies have been reported to assess yielding rockbolts’ performances during rockbursts with actual seismic loading. As mentioned above, the impact loading in drop tests might not be able to represent rockburst loading, because there is a complex interaction between seismic waves, rockbolts, and reinforced rock masses during rockbursts with explicit rock detachment and ejection (requiring distinct element method (DEM) or DEM-related hybrid methods). Therefore, the influence of realistic rockburst loading on the performance of yielding rockbolts remains unclear. Hence, it is essential to evaluate the performance of yielding rockbolts during rockbursts using DEM to provide some references to improve and optimize the design of rock supporting in burst-prone grounds.

This study aims to evaluate the performance of yielding rockbolts during rockbursts with actual seismic loading using numerical modeling method. Two types of yielding rockbolts, namely D-bolt and Roofex, are modeled via the “rockbolt” element in a DEM software UDEC after an exact calibration procedure. D-bolt is a typical representative of a type of yielding rockbolts with high strengths and deformation capacity, while Roofex stands for the other type of yielding rockbolts having low strengths but excellent deformation capacity (Fig. 2). The fully resin-grouted rebar bolt is also simulated to demonstrate the differences between yielding and traditional rockbolts. Instead of conventional drop tests, a two-dimensional (2D) model of a deep tunnel in an underground coal mine is built to fully evaluate the performance (e.g., the dynamic capacity of energy-absorption and control of rock damage) of yielding and traditional rockbolts during the simulated rockbursts. The influence of different rockburst magnitudes is also studied.

## Numerical modeling

### Model setup

**Fig. 3:**
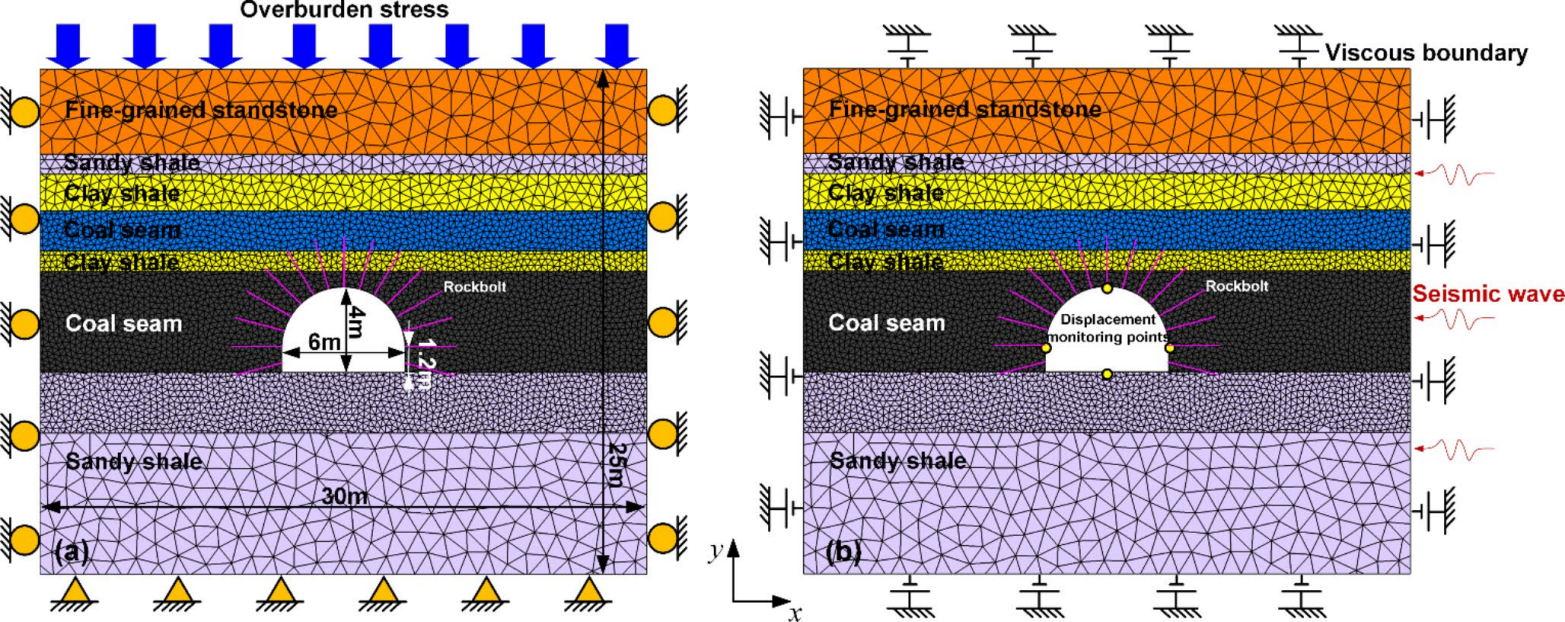
2D numerical model of a deep tunnel. **a** Static stage; **b** Dynamic stage

A widely used 2D DEM software UDEC was used to construct the model of a deep tunnel for conducting the detailed analysis of the effects of yielding rockbolts on controlling rockbursts. The shape of the tunnel cross-section is a semicircular arch, with width and height of 6 m and 4 m, respectively. Noticing that the model size might affect simulation results, two models with different dimensions (small model: 30 m × 25 m, and large model: 60 m × 50 m) were established to examine the effect of model size on the failure depth in surrounding rock masses and the peak stresses in two sidewalls. Table 1 shows that the differences between the two models are minor, e.g., the errors of peak stresses are less than 3%. However, the run time for the initial equilibrium and tunnel excavation in two models are 3.22 and 7.65 h, respectively, when using a regular computer with an Intel i7-3770 CPU at 3.40 GHz (8 cores). The small model can save 57.90% computation costs and is chosen as the final model to conduct subsequent simulation. Figure 3 shows the geometry of the used model, which is based on the lithology and designed size of a deep tunnel in an underground coal mine.

**Table 1:** Comparison between the simulation results of small and large models

Model	Failure depth (m)				Peak stresses in two sidewalls (MPa)		Run time (hour)
	Roof	Floor	Left rib	Right rib	Left side	Right side	
Large model	1.78	2.19	1.31	1.64	39.56	38.62	7.65
Small model	1.75	1.92	1.32	1.44	39.53	39.57	3.22
Error (%)	–1.69	–12.33	0.76	–12.20	–0.08	2.46	—

A Trigon approach developed by Gao et al. (2015) was used to generate blocks in the model, as this approach can be capable of reproducing the natural fracturing processes (e.g., crack initiation, propagation, and coalescence) of rock masses without adopting complicated constitutive models (Chen et al. 2016; Hu et al. 2020; Stavrou et al. 2019; Yang et al. 2017). In the Trigon approach, a rock mass is represented as an assembly of triangular blocks bonded together by contacts (Gao et al. 2015). The fracturing process can be exhibited either by the sliding or opening of contacts. In the model, the average edge length of the blocks in two coal seams and nearby clay shale between them was set to 0.3 m. The block size with a range of 0.2–0.5 m was sufficiently acceptable to simulate the failure behavior of surrounding rock masses for a 2D model (Chen et al. 2016; Gao et al. 2015; Yang et al. 2017). The average edge length of the blocks in the upper clay shale, sandy shale, and fine-grained sandstone was set to 0.5, 0.5, and 1 m, respectively. The average edge length of the blocks on the floor was set to 0.3 and 1 m. A graded increasing edge length of blocks can avoid the resulting loss of simulation accuracy and enhance the calculation’s reliability. The upper boundary of the model was free and a vertical stress of 24.3 MPa (assume the unit weight of overburden is 0.027 MN/m^3^ and the buried depth is 900 m) was applied to the upper boundary to simulate the overburden weight. The roller constraints were applied on lateral boundaries, and the bottom boundary was fixed during the static stage (Fig. 3a). The ratio of horizontal to vertical stress (*K*) was assumed to be one since the hydrostatic stress state is a general situation of the in situ stress in many deep excavations (Dai et al. 2021).

Generally, rockburst can be classified into two types: remotely triggered and self-initiated (Kaiser et al. 1996). Studies have shown that many rockbursts in mining environments are caused by the combination of a remote seismic event triggered by large-scale mining activities and high static stress, while the primary source of rockbursts is the rock mass itself around the tunnel in civil engineering projects (Diederichs 2018; Mutke et al. 2015). This research specifically focuses on the evaluation of the performance of yielding rockbolts during remotely triggered rockbursts. A seismic wave caused by the nearby fault slip was assumed to be the dynamic load source to trigger a rockburst occurring in the tunnel (Gao et al. 2021; Zhang and Nordlund 2019). Mutke et al. 2015 and Kong et al. 2019 reported that the rockburst potential of tunnels has a positive correlation with the peak particle velocity (PPV) of seismic waves and buried depths. The statistical data of rockbursts suggest that PPVs were mainly in the range of 0.05 m/s to 1.0 m/s, and rockbursts are usually related to seismic waves characterized by low frequencies from 10 to 30 Hz (Mutke 2016; Mutke et al. 2009). Therefore, two PPVs of 0.2 m/s and 0.8 m/s were adopted to simulate different dynamic loads (*σ*_n_ = 2(*ρC*_p_)*v*, Itasca 2020). The frequency is assumed to be 20 Hz and the busy time is 120 ms (a vibration period plus a quiet time of 70 ms). The seismic waveform was simplified to be a sine wave since any complex stress wave can be obtained by the Fourier transform of several simple sine waves (Liu 2017). A series of seismic waves were applied to the right boundary of the model to investigate the dynamic responses of the tunnel. The boundary conditions (e.g., fixed boundaries) used in the static stage can cause the reflection of outward propagating waves back into the model and do not allow the necessary energy radiation. Thus, the viscous boundary developed by Lysmer and Kuhlemeyer (1969) was used in the dynamic calculation (Fig. 3b). A recommended Rayleigh damping of 0.5% was applied (Itasca 2020). This value is suitable for many dynamic analyses that involve large block deformation or large joint displacement.

### Constitutive model and rock mass properties

The properties of rock masses (see Table 2) around the tunnel were obtained according to the laboratory tests of intact rock pieces (following International Society for Rock Mechanics (ISRM) recommended standards, Fairhurst and Hudson 1999) and the generalized Hoek-Brown criterion (Marinos and Hoek 2000) using the Geological Strength Index (GSI) system to evaluate rock mass qualities (Małkowski and Ostrowski 2019; Małkowski et al. 2017; Szott et al. 2018). The uniaxial compressive strength (UCS) and deformation modulus of rock masses were estimated from the following equations (Hoek et al. 2002; Hoek and Diederichs 2006):

1 $${\sigma }_{\text{c}\text{m}}={\sigma }_{\text{c}\text{i}}\frac{\left({m}_{\text{b}}+4s-a\left({m}_{\text{b}}-8s\right)\right){\left(\frac{{m}_{\text{b}}}{4+s}\right)}^{as-1}}{2\left(1+a\right)(2+a)}$$

2 $${E}_{\text{m}}={E}_{\text{i}}\left(0.02+\frac{1-D/2}{1+{e}^{\left(\left(60+15D-\text{G}\text{S}\text{I}\right)/11\right)}}\right)$$

where *D* is a factor that depends upon the degree of disturbance to which the rock mass has been subjected by blast damage and stress relaxation. In this study, the value of *D* is assumed to be zero considering that the mechanical tunneling results in minimal disturbance to confined rock masses (Hoek et al. 2002). The calculated results of UCS and deformation modulus of rock masses are also summarized in Table 2.

**Table 2:** Physical and mechanical parameters of rock masses

Lithology	Constant					Intact rock					Rock mass	
	*m*_i_	*m*_b_	*s*	*a*		*ρ* (kg/m^3^)	*σ*_ci_ (MPa)	*E*_i_ (GPa)	*v*		*σ*_cm_ (MPa)	*E*_m_ (GPa)
Coal	17	1.729	0.0008	0.5		1300	9.3	1.86	0.30		2.50	0.23
Clay shale	9	1.327	0.0022	0.5		2500	29.0	5.62	0.31		7.93	1.26
Fine-grained sandstone	17	2.851	0.0039	0.5		2580	90.0	9.52	0.26		24.53	2.92
Sandy shale	12	1.877	0.0031	0.5		2530	26.0	5.23	0.25		7.11	1.42
Gritty clay shale	8	1.192	0.0022	0.5		2440	47.5	6.98	0.32		13.03	1.56

Notes: *m*_i_ is a material constant for intact rocks. *m*_b_, *s*, and *a* are constants for rock masses. *ρ* is the bulk density of intact rocks. *σ*_ci_ is the UCS of intact rocks. *E*_i_ is the Young’s modulus of intact rocks. *v* is the Poisson’s ratio of intact rocks. *σ*_cm_ is the UCS of rock masses and *E*_m_ stands for the deformation modulus of rock masses

**Fig. 4:**
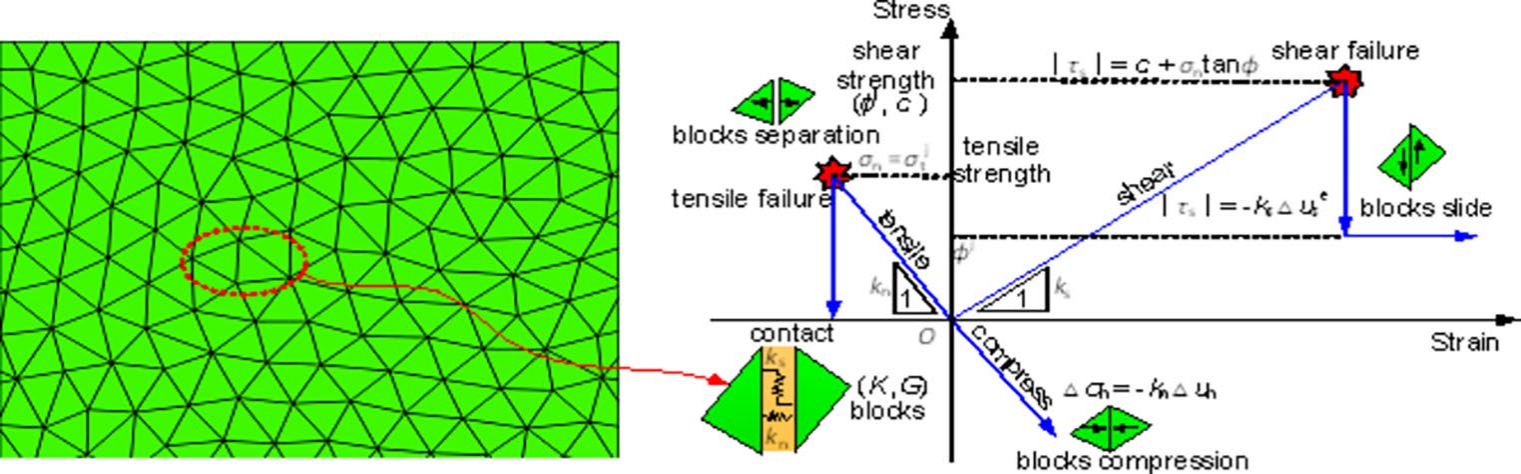
Constitutive behavior of contacts (after Yang et al. 2017)

The elastic constitutive model was chosen for blocks that are composed of finite-difference zones. The coulomb slip model was used for contacts. The constitutive behavior of contacts is shown in Fig. 4. A spring-rider simulates the behavior of contacts, and the model deformation occurs when the contact stress is smaller than the contact strength, which is governed by the elastic modulus of blocks and contact stiffness; contact failure occurs when the stress exceeds its shear or tensile strength, and then blocks will slide or separate with each other (Chen et al. 2016).

**Fig. 5:**
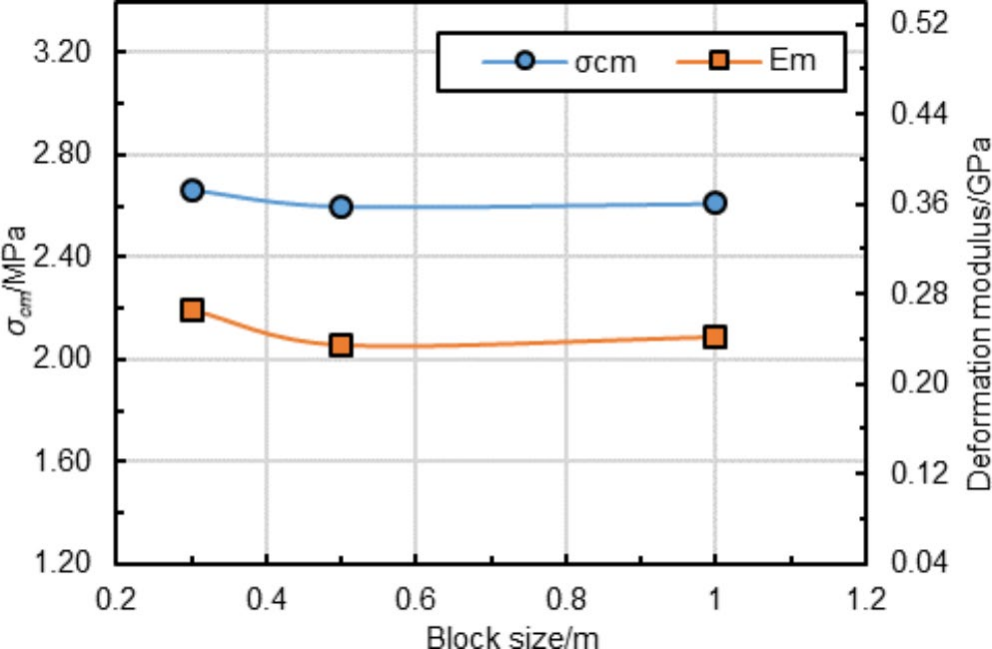
Influence of different block sizes on simulated material properties. (The block edge length of 0.3 m, 0.5 m, and 1 m were used in the sensitivity study)

**Fig. 6:**
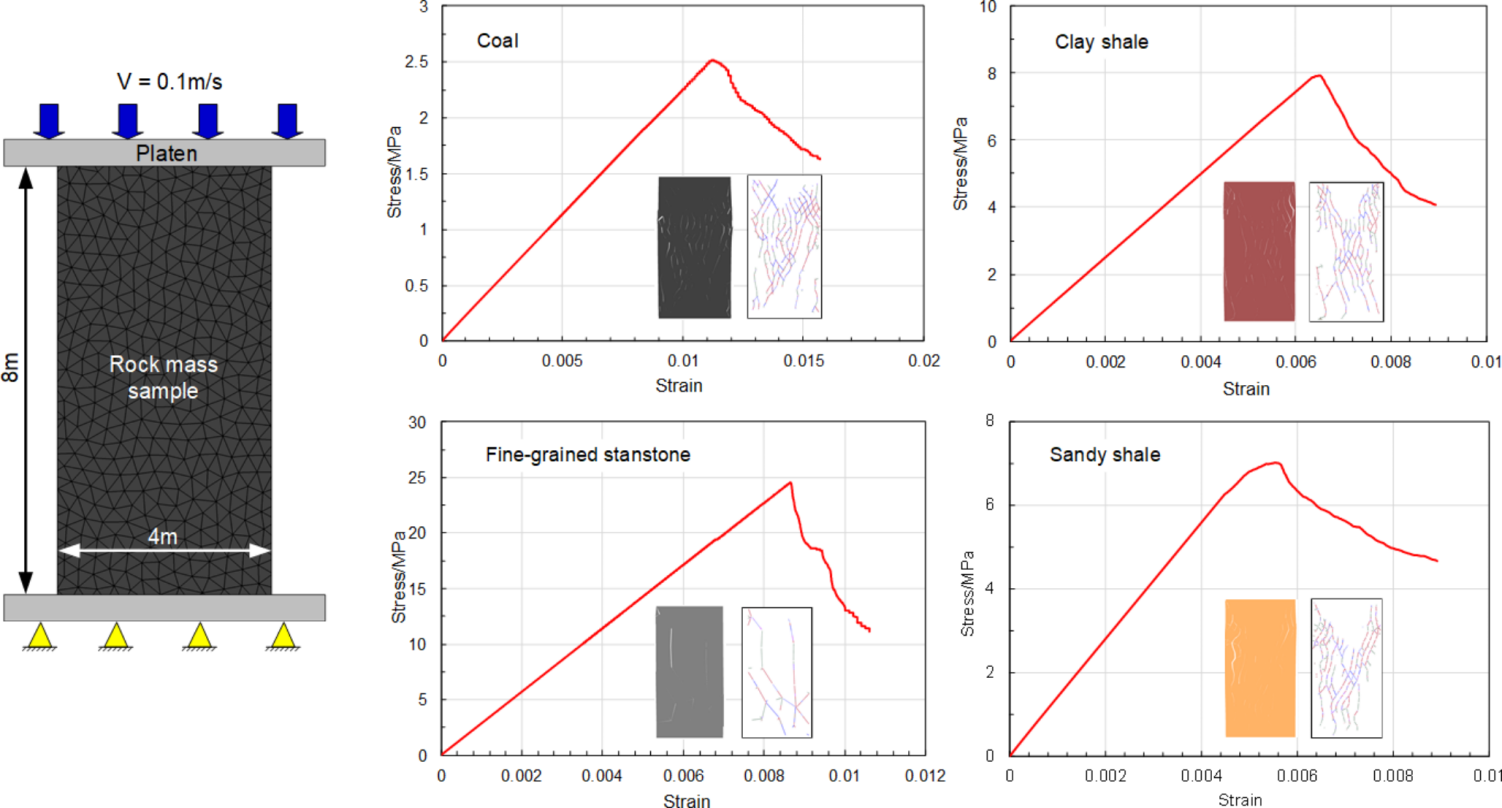
Simulated failure modes and stress-strain curves of rock mass samples

**Table 3:** Calibrated micro parameters of rock masses in the model

Lithology	Block properties				Contact properties				
	*ρ* (kg/m^3^)	*K* (GPa)	*G* (GPa)		*k*_n_ (GPa/m)	*k*_s_ (GPa/m)	*c*^*j*^ (MPa)	*φ*^*j*^ (°)	*σ*_*t*_^*j*^ (MPa)
Coal	1300	0.16	0.09		18.7	7.5	0.99	33	0.25
Clay shale	2500	0.85	0.50		108.5	40.6	2.96	35	0.79
Fine-grained sandstone	2580	1.91	1.17		69.4	27.8	8.11	36	2.15
Sandy shale	2530	0.94	0.57		113.3	45.3	2.95	36	0.85

In the Trigon approach, the deformation and failure of rock masses depend on the properties of blocks and contacts (Chen et al. 2016; Gao et al. 2015). Thus, the micro parameters of blocks and contacts were calibrated against the rock mass properties (Table 2). A series of simulated uniaxial compression tests were conducted to calibrate the micro parameters (Gao et al. 2015). To eliminate the effect of block size on simulation accuracy, the calibration model had a large scale (4 m × 8 m) (Yang et al. 2017) and identical block size with the tunnel model. However, there is a problem that different block sizes were employed for the rock strata with the same lithology (e.g., block size of 0.3 and 0.5 m for clay shale, and 0.3 m, 0.5 m, and 1 m for sandy shale), which means that different material parameters might be used even for the same lithology. A sensitivity study was conducted and shown that block size effect on simulated rock mass properties can be negligible (Fig. 5). A displacement loading mode was used in the simulation by applying a constant velocity of 0.1 m/s to the surface of the top platen, and the bottom platen was fixed. This loading rate of 0.10–0.15 m/s is slow enough to avoid the dynamic responses of models because UDEC automatically selects very small time steps (e.g., 10^− 7^ s) in static analysis (Hu et al. 2020; Gao et al. 2019). The initial micro parameters were first assumed based on the macro parameters of rock masses. Then, the modeling of uniaxial compression tests was conducted iteratively with the adjustment of micro parameters until the simulated results were consistent with the targeted material properties. Tan and Konietzky (2014) gives a detailed calibration process of micro parameters. The simulated failure modes and stress-strain curves of rock mass samples are shown in Fig. 6. The calibrated micro parameters of rock masses are listed in Table 3. The errors between the targeted and simulated deformation modulus and UCS are all less than 3% (Table 4), suggesting that the targeted values agree well with calibrated rock mass parameters. Thus, the calibrated micro parameters in Table 3 could be used for further numerical analysis to evaluate the performance of yielding rockbolts during rockbursts.

**Table 4:** Comparison between the targeted and simulated rock mass parameters

Lithology	*E*_m_ (GPa)				UCS (MPa)		
	Target	Simulation	Error (%)		Target	Simulation	Error (%)
Coal	0.23	0.226	0.09		2.50	2.51	0.48
Clay shale	1.26	1.234	-1.82		7.93	7.91	-0.29
Fine-grained sandstone	2.92	2.852	-2.48		24.53	24.52	-0.05
Sandy shale	1.42	1.39	-2.11		7.11	7.02	-1.27

### Properties of rockbolts

#### Introduction of rockbolt element

**Fig. 7:**
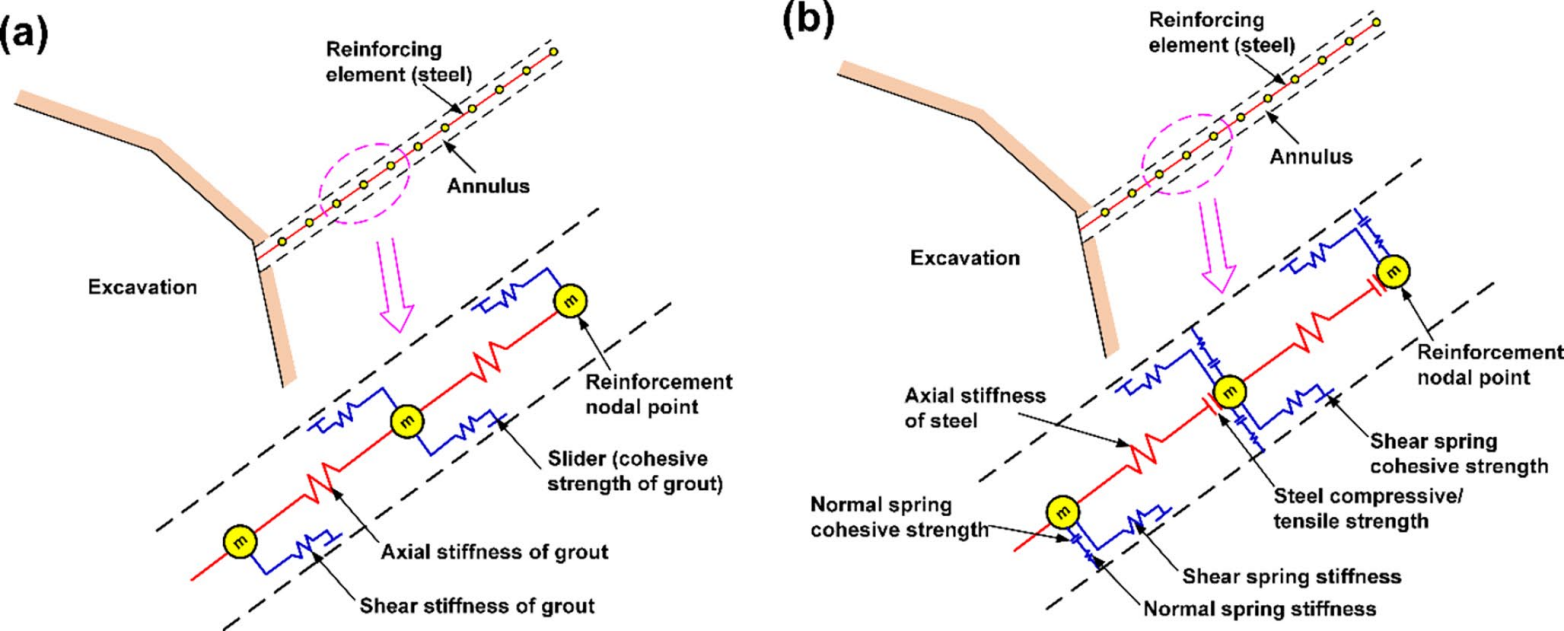
Conceptual mechanical representation of the global reinforcement: **a** “cable” element, which accounts for shear behavior of the grout annulus, and **b** “rockbolt” element, which accounts for shear behavior of grout annulus and bending resistance of the reinforcement (after Itasca 2020)

In the past, the “cable” element in UDEC was more popular used than the “rockbolt” element to model a mechanically anchored or grouted cable or rockbolt, although both elements can simulate the shearing resistance along their length, which is provided by the shear bond between the grout and either the cable/rockbolt or the host rock (Bahrani and Hadjigeorgiou 2017). This could be owing to more understandable input parameters and the more straightforward calibration process for using the “cable” element. Figure 7 shows the conceptual mechanical representation of “cable” and “rockbolt” elements. It can be seen that both two types of elements are composed of several segments and nodal points located at segment ends. Nevertheless, the “rockbolt” element has both shear and normal coupling springs, which are connectors that transfer forces and motion between the “rockbolt” element and the grid points associated with the block zone, while the “cable” element only has sliders (similar to shear coupling springs). Therefore, the “cable” element provides little resistance to bending, and thus it is more suitable for modeling cable bolts.

**Fig. 8:**
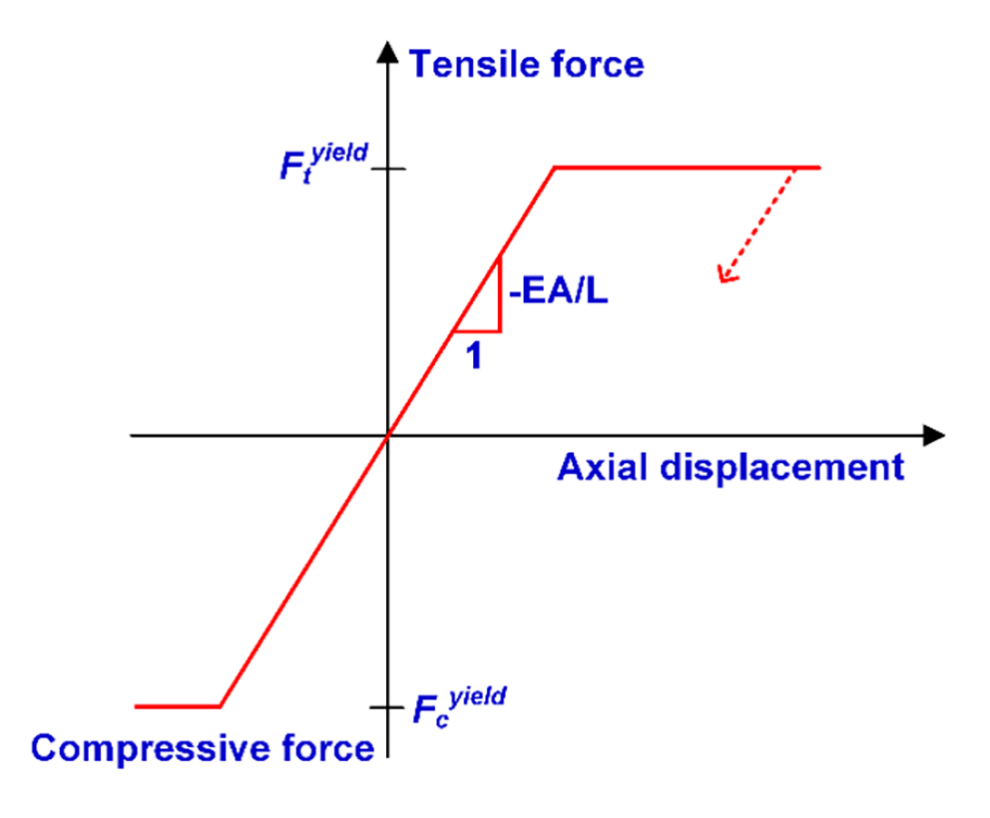
Mechanical behavior of the “rockbolt” element in the axial direction (Itasca 2020)

In contrast, the “rockbolt” element can provide sufficient resistance for shearing and bending, which is appropriate for simulating rockbolts such as rebar bolts (Tomasone et al. 2020). The other strength of the “rockbolt” element is that it can explicitly model the rockbolt breakage according to a user-defined tensile failure strain limit (Itasca 2020), providing a more accurate and realistic approach to reproduce rockbolt performances. Thus, the “rockbolt” element was used in this study to simulate the mechanical behavior of both yielding and conventional rockbolts. The “rockbolt” element has a linearly elastic material behavior in UDEC that it can yield in both tension and compression in the axial direction (Fig. 8). The incremental axial force in a “rockbolt” element, △*F*_*t*_, can be obtained by the calculation of the incremental axial displacement:

3 $${\varDelta F}_{t}=-\frac{EA}{L}\varDelta {u}^{t}$$

where △*u*^*t*^ = △*u*_*i*_*t*_*i*_ = △*u*_1_*t*_1_ + △*u*_2_*t*_2_ = (*u*_1_^[*b*]^ - *u*_1_^[*a*]^)*t*_1_ + (*u*_2_^[*b*]^ – *u*_2_^[*a*]^)*t*_2_; *u*_1_^[*b*]^, *u*_1_^[*a*]^, etc. are the displacements at the bolt nodes associated with each “rockbolt” element. Subscript 1 and 2 represent the x-direction and y-direction, respectively; the superscripts [*a*], [*b*] stand for bolt nodes. The direction cosines *t*_1_, *t*_2_ refer to the tangential (axial) direction of the “rockbolt” element.

**Fig. 9:**
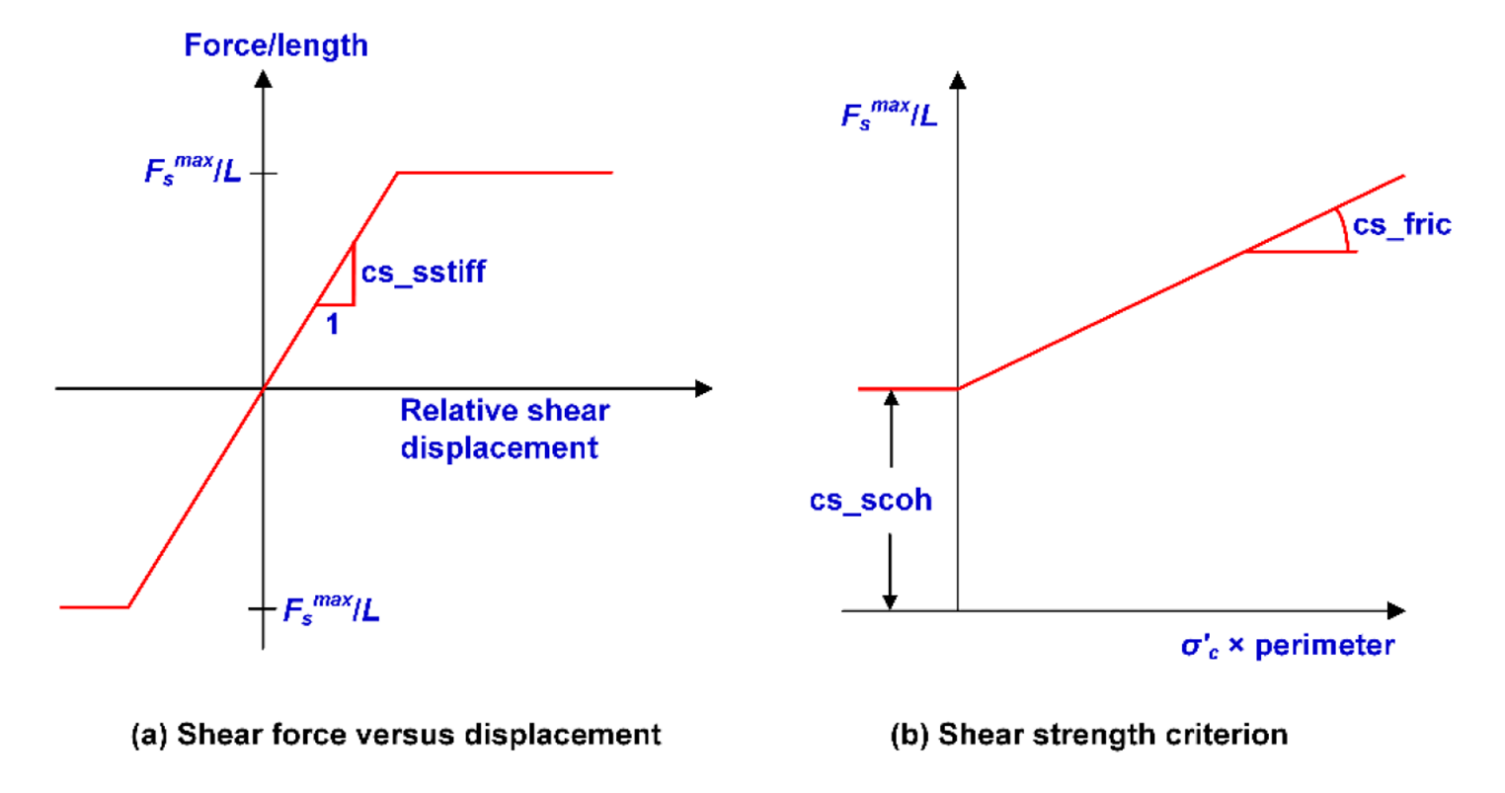
Mechanical behavior of shear coupling spring for the “rockbolt” element (Itasca 2020)

The shear and normal behavior of the “rockbolt” element were briefly introduced in this study. The shear behavior of the rockbolt/gridpoint interface is represented as a spring-slider system at the rockbolt nodal points. This behavior during relative displacement can be described numerically by the coupling spring shear stiffness (*cs*_sstiff_ in Fig. 9a):

4 $$\frac{{F}_{\text{s}}}{L}={cs}_{\text{s}\text{s}\text{t}\text{i}\text{f}\text{f}}\left({u}_{\text{p}}-{u}_{\text{m}}\right)$$

where, *F*_s_ represents the shear force that develops in the shear coupling spring (e.g., along with the interface between the rockbolt element and the gridpoint); *cs*_sstiff_ is the coupling spring shear stiffness; *u*_p_ is the axial displacement of the rockbolt; *u*_m_ is the axial displacement of the medium (soil or rock); and *L* is the contributing element length.

The maximum shear force that can be developed along the rockbolt/gridpoint interface is a function of the cohesive strength of the interface and the stress-dependent frictional resistance along with the interface (Fig. 9b). The following equation can be used to determine the maximum shear force per length of the rockbolt:

5 $$\frac{{F}_{\text{s}}^{\text{m}\text{a}\text{x}}}{L}={cs}_{\text{s}\text{c}\text{o}\text{h}}+{\sigma }_{\text{c}}^{{\prime }}\times \text{tan}\left({cs}_{\text{s}\text{f}\text{r}\text{i}\text{c}}\right)\times perimeter$$

where, *cs*_scoh_ is the cohesive strength of the shear coupling spring; *σ’*_c_ is the average effective confining stress perpendicular to the “rockbolt” element; *cs*_sfric_ is the friction angle of the shear coupling spring, and perimeter is the exposed perimeter of the element.

**Fig. 10:**
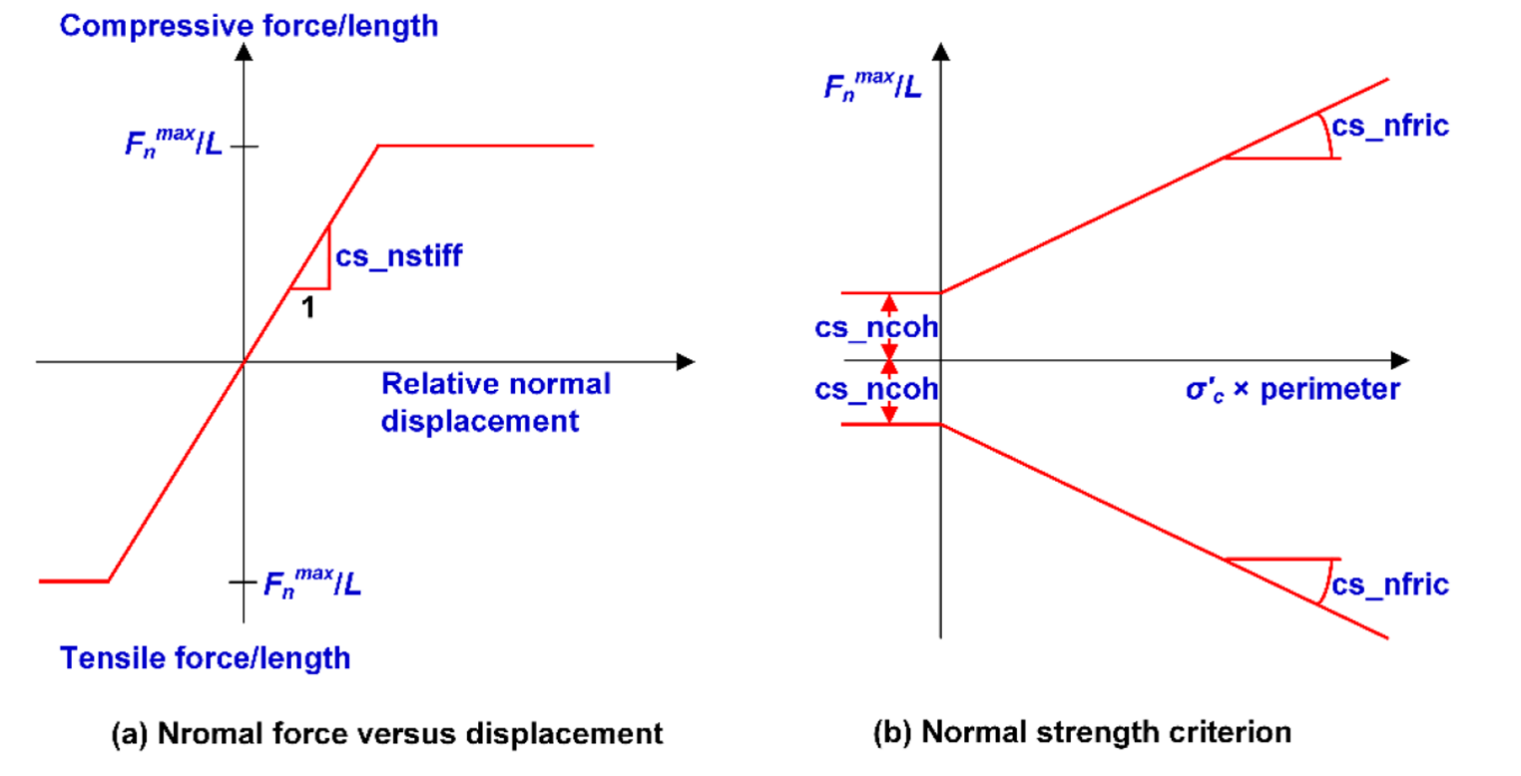
Mechanical behavior of normal coupling spring for the “rockbolt” element (Itasca 2020)

The normal behavior of the rockbolt/gridpoint interface is represented by a linear spring with a limiting normal force that is dependent on the direction of movement of the rockbolt node. The expected behavior during the relative normal displacement between the rockbolt nodes and the gridpoint can be described numerically by the coupling spring normal stiffness (*cs*_nstiff_ in Fig. 10a):

6 $$\frac{{F}_{\text{n}}}{L}={cs}_{\text{n}\text{s}\text{t}\text{i}\text{f}\text{f}}\left({u}_{\text{p}}^{\text{n}}-{u}_{\text{m}}^{\text{n}}\right)$$

where, *F*_n_ represents the normal force that develops in the normal coupling spring (e.g., along with the interface between the rockbolt element and the gridpoint); *cs*_nstiff_ is the coupling spring normal stiffness; *u*_p_^n^ is the displacement of the rockbolt perpendicular to the axial direction of the rockbolt; *u*_m_^n^ is the displacement of the medium (soil or rock) perpendicular to the axial direction of the rockbolt, and *L* is the contributing element length.

A limiting normal force can be prescribed to stimulate the localized three-dimensional effect of the rockbolt pushing through the grid (e.g., the soil being squeezed around a single rockbolt). The limiting normal force is a function of normal cohesive strength and a stress-dependent frictional resistance between the rockbolt and the gridpoint (Fig. 10b). The following equation can be used to determine the maximum normal force per length of the rockbolt:

7 $$\frac{{F}_{\text{n}}^{\text{m}\text{a}\text{x}}}{L}={cs}_{\text{n}\text{c}\text{o}\text{h}}+{\sigma }_{\text{c}}^{{\prime }}\times \text{tan}\left({cs}_{\text{n}\text{f}\text{r}\text{i}\text{c}}\right)\times perimeter$$

where, *cs*_ncoh_ is the cohesive strength of the normal coupling spring, which is dependent on the direction of loading; *σ’*_c_ is the average effective confining stress perpendicular to the rockbolt element; *cs*_nfric_ is the friction angle of the normal coupling spring, and perimeter is the exposed perimeter of the element.

#### Calibration of rockbolt properties

The hypothesis of this study is that the drop test could not be representative of rockburst loading and the real performance of yielding rockbolts is complex, which should be evaluated during rockbursts with actual seismic loading. The pull-out test is a well-recognized test and it can represent the static load-displacement characteristics of rockbolts before rockbursting and the performance of rockbolts during rockbursts will be initially confirmed by in situ observations and others’ simulation results. Hence, only the simulated pull-out tests were conducted to calibrate the input parameters of the “rockbolt” element with the comparison of the laboratory test results from Charette and Plouffe (2007), Stillborg (1994), and Li (2011). The size of the model is 2 m ×1 m, and the bolt length is 2 m. This model size is almost identical to that used by Bahrani and Hadjigeorgiou (2017). The model has a Young’s modulus of 7.5 GPa and a Poisson’s ratio of 0.25 to represent an elastic rock mass, because it has been confirmed that the elastic properties of the rock mass do not influence the load-displacement response of the “rockbolt” element (Tomasone et al. 2020) which can significantly save computation time. The rockbolt was divided into 40 segments and 41 nodes to ensure that at least one node falls into each block zone (Bahrani and Hadjigeorgiou 2017). The upper boundary of the model was free, and a vertical upward velocity of 0.08 m/s was applied to the end node of the bolt to simulate a pull action (Zhu et al. 2020). The roller constraints were applied on the side boundaries and the bottom boundary. A function was developed using the FISH language (built-in programming package) in UDEC to monitor the axial force of the last segment of the rockbolt.

**Fig. 11:**
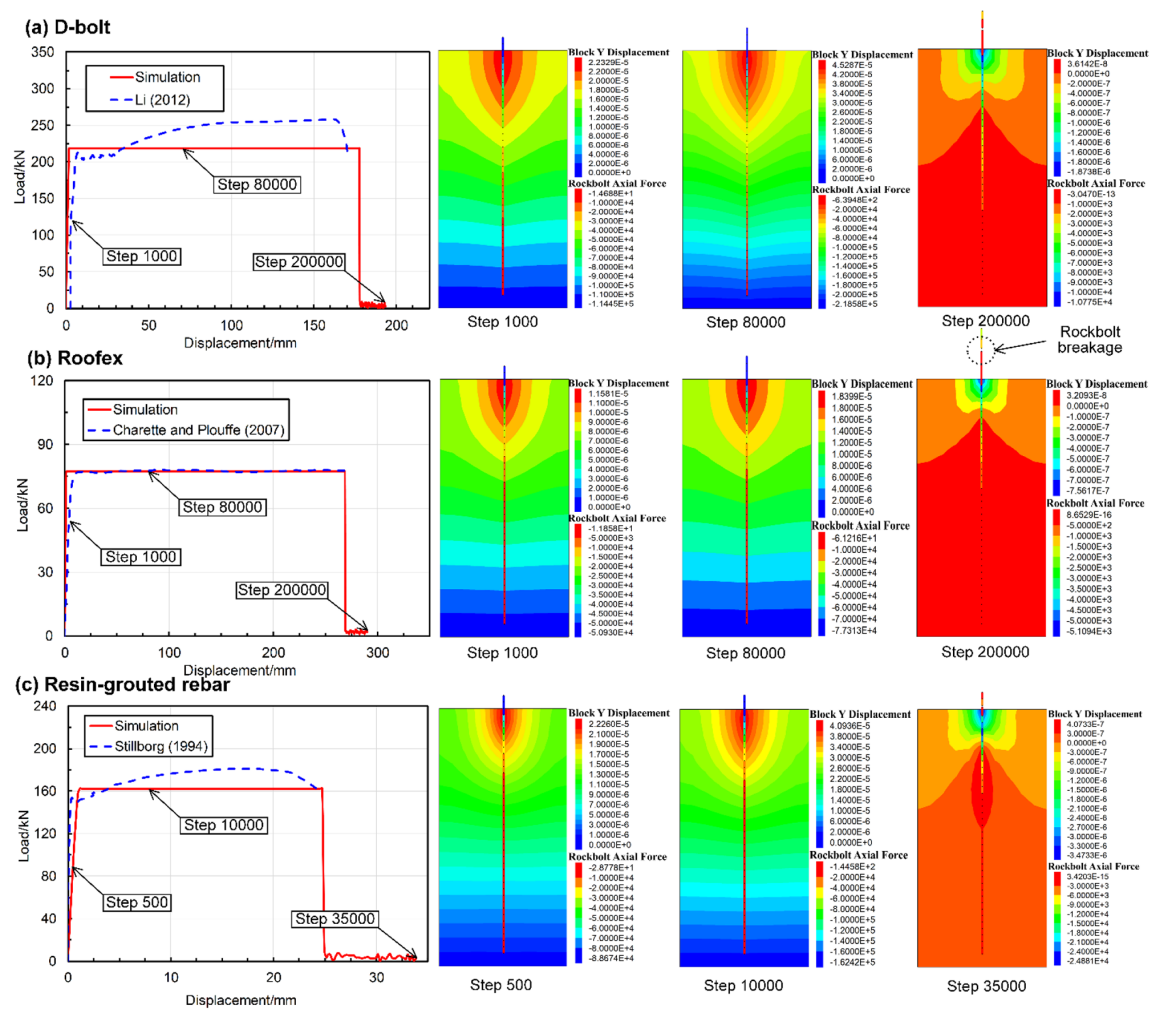
Simulated load-displacement curves and axial forces of rockbolts and deformation of rock masses. (Rockbolt axial force in N and block Y displacement in m)

**Table 5:** Calibrated input parameters of rockbolts

Rockbolt type	Cross-sectional area (m^2^)	Moment of inertia (m^4^)	Perimeter of borehole (m)	Density (kg/m^3^)	Elastic modulus (GPa)	Tensile yield strength (kN)	Tension failure strain	Shear coupling spring stiffness (GN/m/m)	Shear coupling spring cohesion (kN/m)	Shear coupling spring friction angle (°)
Resin-grouted rebar	3.14 × 10^− 4^	7.85 × 10^− 9^	0.08	7500	200	517	0.33	0.31	400	45
D-bolt	3.80 × 10^− 4^	1.15 × 10^− 8^	0.10	7500	200	575	1.36	0.29	438	45
Roofex	1.23 × 10^− 4^	1.20 × 10^− 9^	0.08	7500	200	630	1.66	0.21	353	45

The modeling of pull-out tests was conducted iteratively to adjust input parameters (e.g., tensile yield strength, tension failure strain, shear coupling spring stiffness, and shear coupling spring cohesion, Itasca 2020) until the simulated results were consistent with the targeted properties of rockbolts. Other input parameters (e.g., the diameter, length, density, and elastic modulus of rockbolts) are the same as that used in laboratory tests. The simulated load-displacement curves and axial force of rockbolts and the block displacement are shown in Fig. 11. The calibrated input parameters of rockbolts are listed in Table 5. The applied load is axial in an ideal pull-test as simulated in this study. Thus, the parameters regarding resistance to bending are not employed. The errors between the targeted and simulated ultimate load, rupture displacement, and static energy-absorption capacity of rockbolts are less than 5% (Table 6), indicating that the targeted values agree well with calibrated input parameters. Thus, the calibrated parameters in Table 5 could be used to further the numerical analysis of the performance of yielding and conventional rockbolts (Zhang and Nordlund 2019). However, it should be noted that the sliding or extraction of Roofex was not simulated explicitly in the pull-out test, and its energy-absorption mechanism was simplified to the deformation or stretch of bolt shanks. This equivalent approach could be regarded as a relatively good selection at this stage since the complexity of simulating bolt sliding was ignored, and the time cost was thus significantly reduced.

**Table 6:** Comparison between the targeted and simulated rockbolt properties

Rockbolt type	Ultimate load (kN)				Rupture displacement (mm)				Static energy-absorption capacity (kJ)		
	Laboratory test	Simulation	Error (%)		Laboratory test	Simulation	Error (%)		Laboratory test	Simulation	Error (%)
Resin-grouted rebar	162	162	0.0		24.1	24.9	3.3		4.15	3.96	-4.6
D-bolt	212	219	3.3		170	178	4.7		40.23	38.65	-3.9
Roofex	77.6	77.3	-0.4		274	269	-1.8		20.94	20.71	-1.1

### Simulation process and schemes

The modeling of the performance of yielding rockbolts during rockbursts was performed with the following stages and schemes.

Stage I (static stage): The in-situ stress field was applied to the model, and the geostatic equilibrium was achieved. Then, the tunnel was excavated by deleting the blocks. Adequate calculation steps were run to ensure the gradual and slow release of surrounding rock stresses (Gao et al. 2015). The installation of rockbolts was conducted immediately after the excavation of the tunnel.

Stage II (dynamic stage): The dynamic mode was activated in this stage. Rockbursts having different magnitudes were produced by exerting a series of seismic waves with varied PPVs (0.2 m/s and 0.8 m/s, representing weak and strong rockbursts, Mutke et al. 2015). The pattern layout of rockbolts in the tunnel is shown in Fig. 2. The roof and two ribs of the tunnel were supported by 15 rockbolts in total, while the floor remained unsupported, as is a common practice. The roof and rib bolts have a length of 2.5 m and row spacing of 0.7 m. The out-of-plane spacing of rockbolts is one meter by setting the “spacing” parameter in UDEC. Besides, D-bolt, Roofex, and fully resin-grouted rebar were simulated in each scheme.

## Analysis of simulation results

### Displacement and velocity analysis

**Fig. 12:**
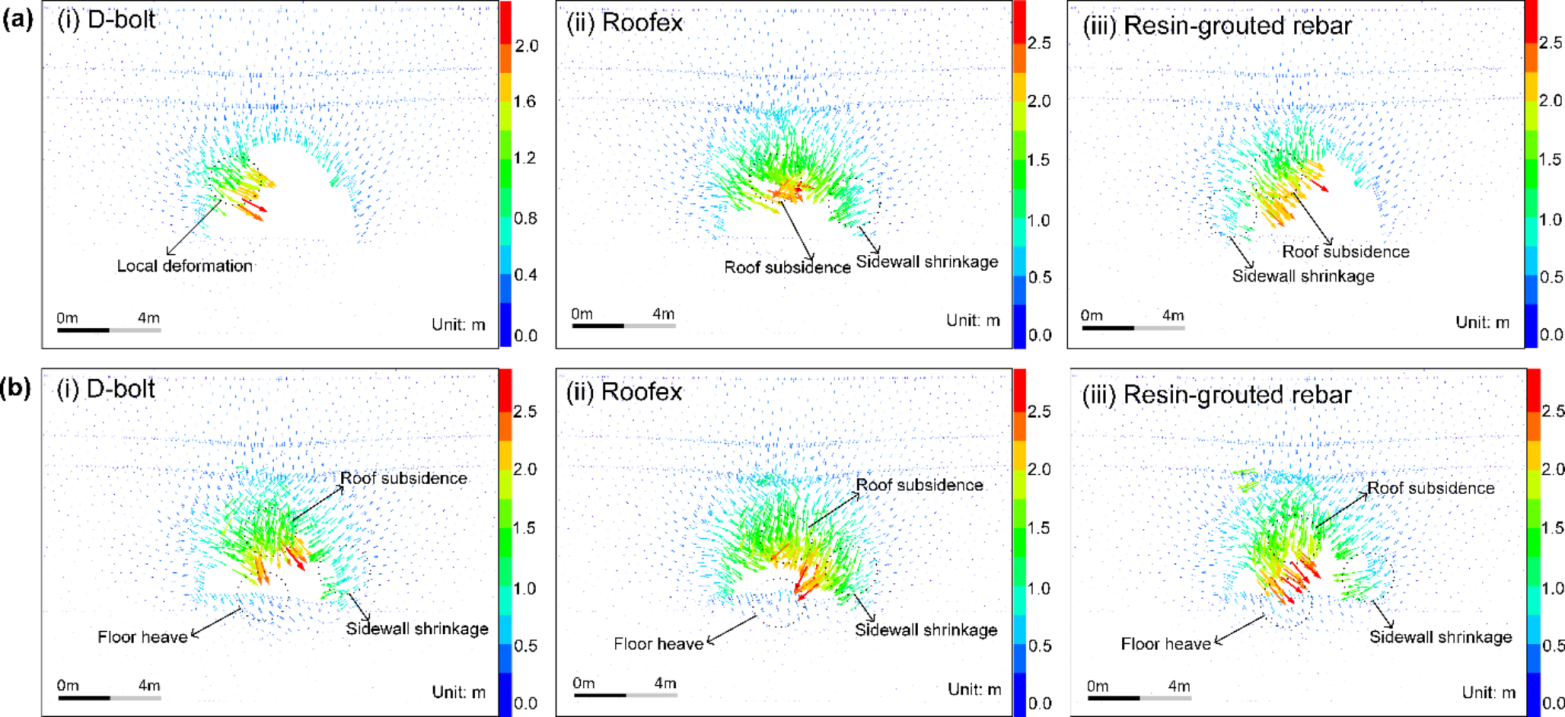
Simulated displacement vectors of the surrounding rock masses along the tunnel supported by different types of rockbolts. **a** PPV = 0.2 m/s; **b** PPV = 0.8 m/s

**Fig. 13:**
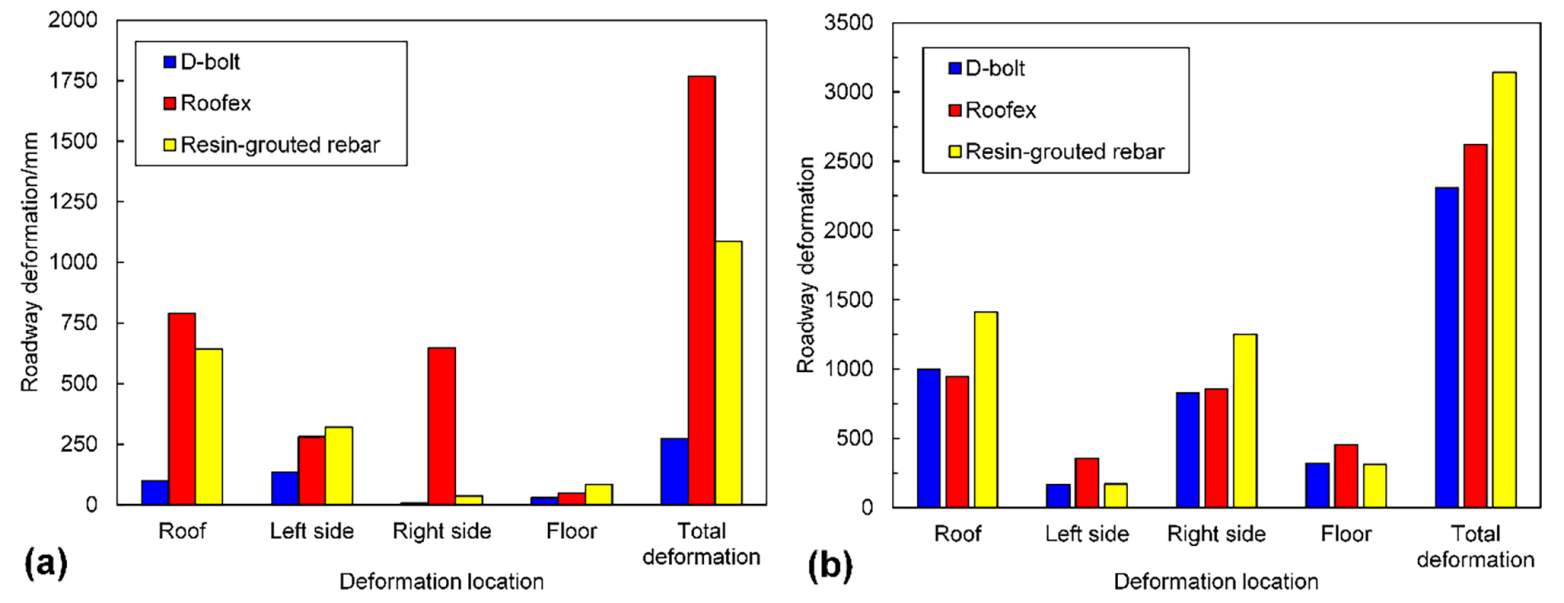
Comparison of the deformation of the tunnel supported by different types of rockbolts. **a** PPV = 0.2 m/s; **b** PPV = 0.8 m/s

The simulated displacement patterns of the tunnel supported by different types of rockbolts are shown in Fig. 12. When the PPV is 0.2 m/s (see Fig. 12a), large deformation only occurs in a local tunnel area that D-bolts support. In contrast, noticeable roof subsidence and sidewall shrinkage are observed when the tunnel is supported with Roofex and resin-grouted rebar. When the PPV is 0.8 m/s, the deformation of tunnel surrounding rock masses aggravates due to more significant dynamic stresses. In addition to roof subsidence and sidewall shrinkage, severe floor heaving occurs in all three support schemes. This phenomenon agrees with many facts that significant floor heaving is often observed in rockburst events with high seismic magnitudes (Mutke et al. 2009; Prusek and Masny 2015). To further investigate the effects of different types of rockbolts on controlling rockbursts, four monitoring points were arranged at the roof, floor, and two sidewalls of the tunnel to record the tunnel deformation induced by rockbursts (Fig. 2). The comparison of the tunnel deformation in three support schemes is shown in Fig. 13. When the PPV is 0.2 m/s (Fig. 13a), the tunnel supported by D-bolts suffers minor deformation (only 273 mm in total). However, the total deformations of the tunnel supported with Roofex and resin-grouted rebar are 1767 and 1086 mm, respectively, which are 6.47 and 3.98 times that of the tunnel supported by D-bolts. The most severe deformation is found when Roofex supports the tunnel. This is because the Roofex possesses the lowest strength (77 kN) compared to the D-bolt (219 kN) and resin-grouted rebar (162 kN). Thus, the Roofex fails to restrain the large deformation induced by rockbursts. When the PPV is 0.8 m/s (Fig. 13b), the tunnel deformations in three support schemes all experienced significant growth. For instance, the total deformation of the tunnel supported by D-bolts increases from 273 to 2310 mm, with a growth rate of 746%.

**Fig. 14:**
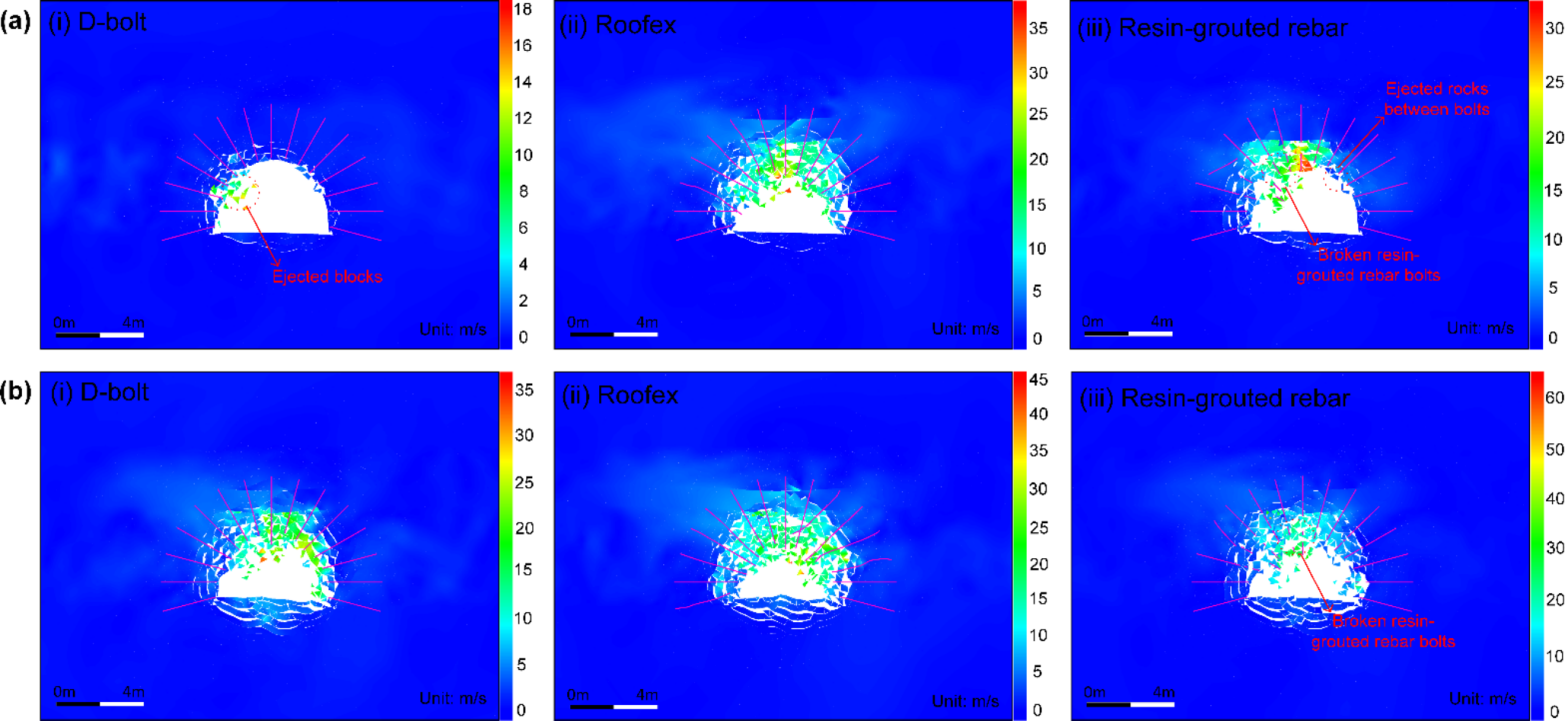
Simulated velocity distribution of the surrounding rock masses along the tunnel supported by different types of rockbolts. **a** PPV = 0.2 m/s; **b** PPV = 0.8 m/s

**Fig. 15:**
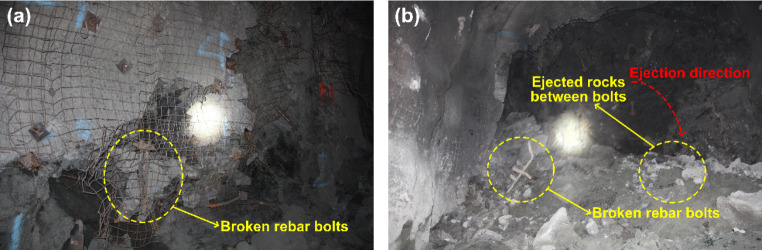
In-situ observations of broken rebar bolts after rockbursts in deep tunnels in Canada (photographs taken by authors)

The tunnel deformation in this scenario is still the least compared to the other two support schemes. However, it can be found that the tunnel supported by resin-grouted rebar rather than Roofex suffers the most severe deformation. Although the resin-grouted rebar has relatively high strength, its elongation rate is low and easy to break during dynamic shocks. As shown in Fig. 14a (iii), b (iii), many resin-grouted rebar bolts are broken during the rockburst, and therefore, they are unable to control rapid rock bulking or ejection effectively. Some in situ observations can confirm this phenomenon (Fig. 15). In summary, the tunnel profile continuously shrinks with increasing seismic magnitudes. All three types of rockbolts fail to control the large deformation induced by dynamic stresses during the violent rockburst (PPV = 0.8 m/s).

**Fig. 16:**
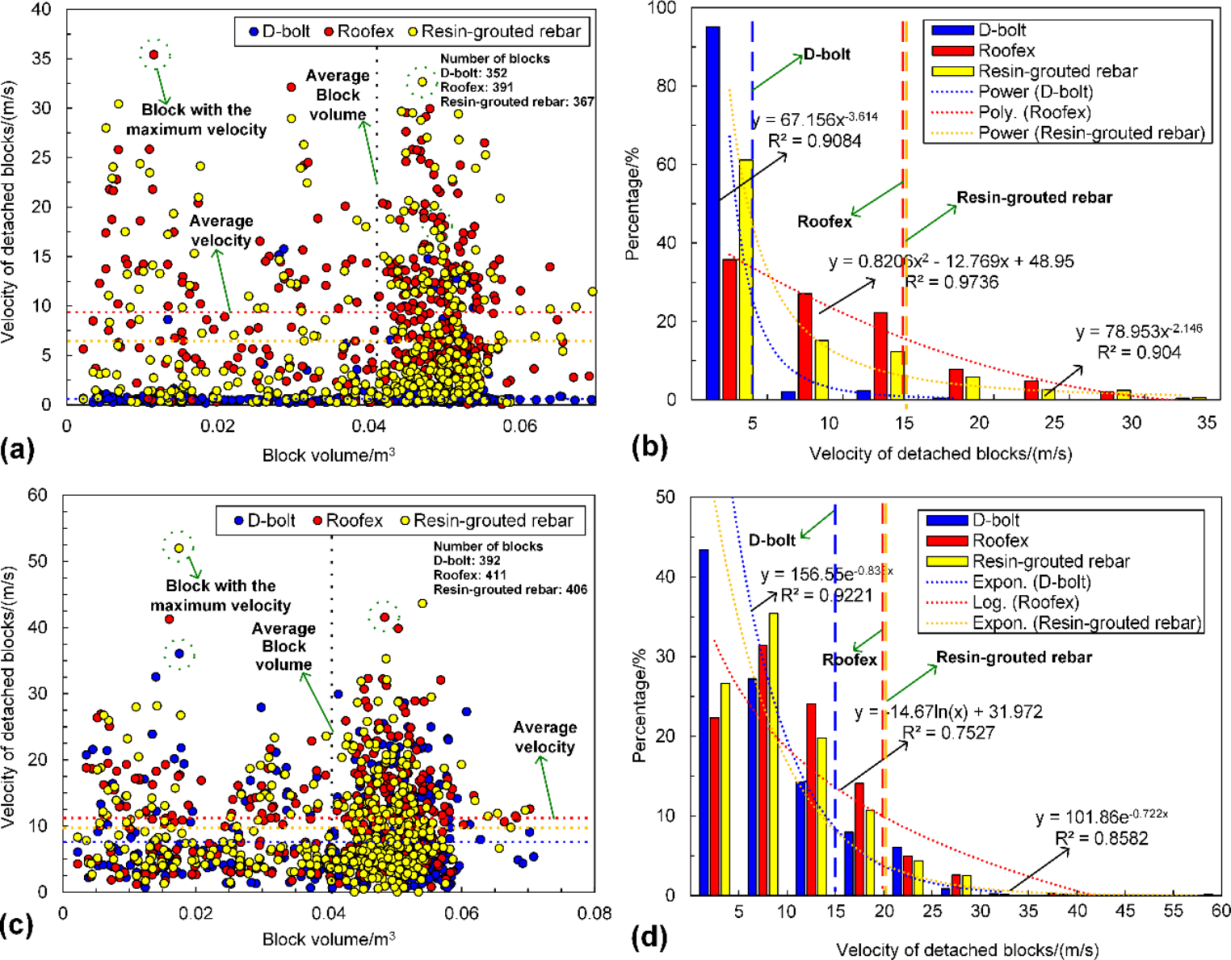
**a** and **c** is the velocity of all detached blocks versus block volume when the PPVs are 0.2 m/s and 0.8 m/s, respectively; **b** and **d** are the velocity distribution of all detached blocks when the PPV is 0.2 m/s and 0.8 m/s, respectively

The velocity distribution of tunnel surrounding rock masses in three support schemes is shown in Fig. 14. It can be seen from Fig. 14a that only a few rock blocks are ejected from a local zone when the D-bolt is adopted. For the tunnel supported by Roofex and resin-grouted rebar, much more rock blocks are ejected from the roof and sidewalls. With the increase of PPVs, the range of the region with high velocities grows (see Fig. 14b). A large quantity of ejected rock blocks is observed no matter which type of rockbolts is used. To further study the effects of different types of rockbolts on mitigating rockburst damage, a function was developed using FISH language in UDEC to record the velocity and volume of all the detached rock blocks in the model. The statistical analysis results are illustrated in Fig. 16. As shown in Fig. 16a, the average velocity of rock blocks in the tunnel supported by D-bolts is only 1.20 m/s, although a few blocks may have a relatively high velocity (e.g., 10–18 m/s). By comparison, the average velocities of rock blocks in the tunnel supported with Roofex and resin-grouted rebar are 9.07 and 6.65 m/s, respectively. Additionally, the velocity distributions of rock blocks in these two scenarios are more extensive than those in the tunnel using D-bolts. Figure 16b shows that 95.1% of rock blocks in the tunnel supported by D-bolts possesses a velocity lower than 5 m/s, while the velocities of most rock blocks in the other two scenarios (85.1% for Roofex and 88.6% for resin-grouted rebar) are within the range of 0–15 m/s. Many rock blocks focus on the volume range of 0.04–0.055 m^3^ due to the setting of the edge length of blocks (0.3 m) within the tunnel surrounding rock masses. When the PPV is 0.8 m/s (Fig. 16c), the average velocities of rock blocks in three support schemes undergo growth. For example, the average velocity of rock blocks in the tunnel supported by D-bolts increases from 1.20 to 8.34 m/s, with a growth rate of 595%. It is also found that the velocity distributions of rock blocks in three support schemes are more extensive than that in the tunnel during the weak rockburst. As shown in Fig. 16d, 84.8% of rock blocks in the tunnel supported by D-bolts possesses the velocity of 0–15 m/s, while the velocities of most rock blocks in the other two scenarios (91.9% for Roofex and 88.6% for resin-grouted rebar) are within the range of 0–20 m/s. Another finding is that more rock blocks are detached or ejected when the PPV is 0.8 m/s. For instance, the number of detached rock blocks in the tunnel supported by D-bolts increases from 352 to 392, with a growth rate of 11.36%. These results suggest that the rockburst is more violent when the PPV is high and further confirm that supporting three types of rockbolts solely are unable to control violent rockbursts.

### Rockburst damage analysis

Studies have shown that many rock engineering accidents, including rockbursts, are due to the weakening of rock mass strengths resulted from the initiation and development of internal fractures (Chen et al. 2016; Gao et al. 2015; Yang et al. 2017). To investigate the influences of different types of rockbolts on mitigating rockburst damage, the crack distribution, macroscopic failure pattern, and damage degree of the tunnel induced by rockbursts were analyzed. A function was developed using FISH language in UDEC to record the length and number of failed contacts (represent cracks, including tensile and shear failure) in the model. A damage variable was then defined in the self-developed FISH function according to the ratio of the length of failed contacts to the total contact length in the model (Gao et al. 2015):

8 $${D}_{\text{c}}=\frac{{L}_{\text{t}}+{L}_{\text{s}}}{{L}_{\text{c}}}, {D}_{\text{t}}=\frac{{L}_{\text{t}}}{{L}_{\text{c}}}, {D}_{\text{s}}=\frac{{L}_{\text{s}}}{{L}_{\text{c}}}$$

where *D*_c_, *D*_t_, and *D*_s_ are total, tensile, and shear damage degrees, respectively. *L*_c_, *L*_t_, and *L*_s_ are total contact length and the length of failed contacts in tensile and shear failure. Additionally, the severity of rockburst damage can also be evaluated by the volume of failed rocks (Cai 2013). Therefore, the volume of ejected rock blocks is also recorded using another self-developed FISH function in the UDEC model.

**Fig. 17:**
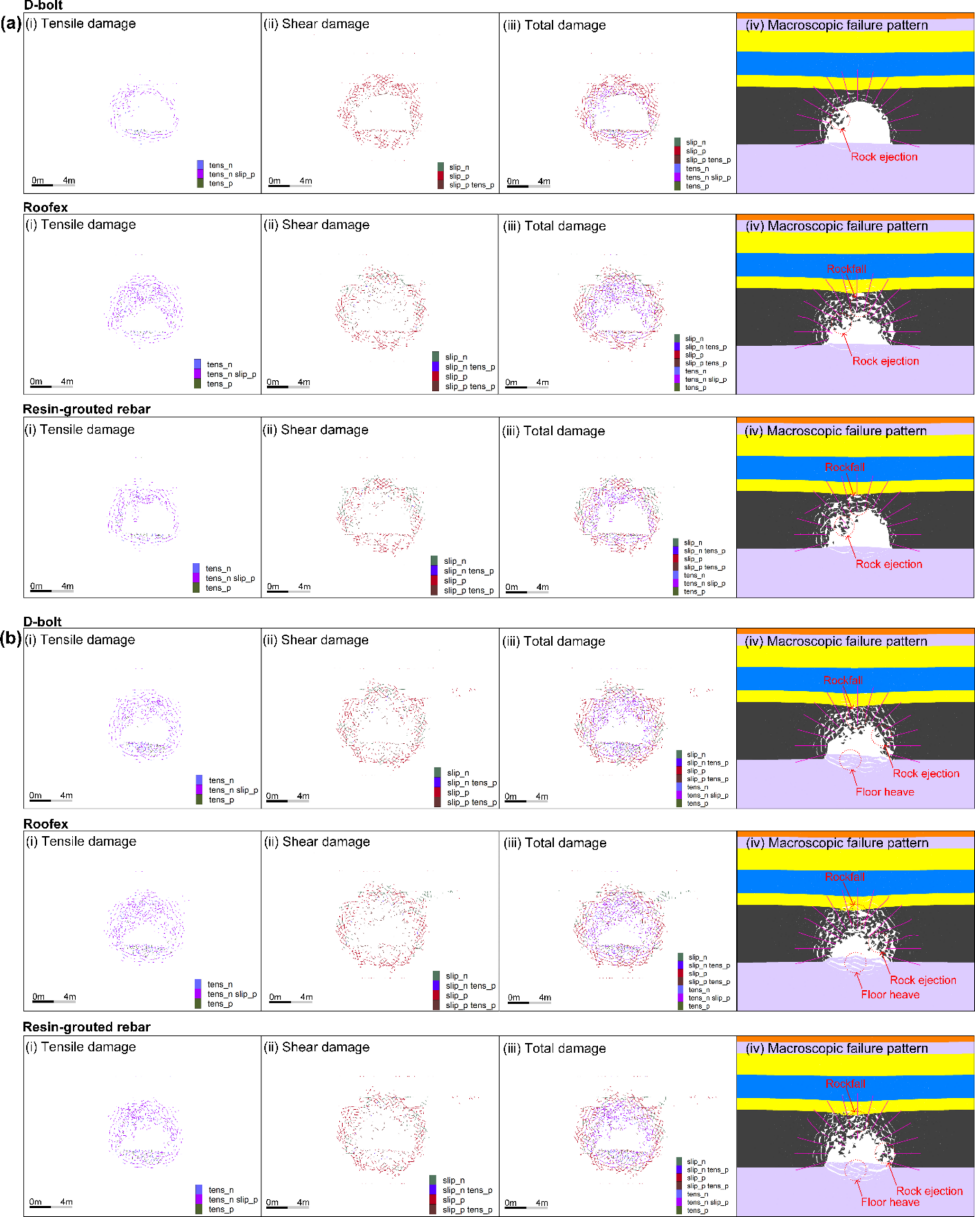
Distribution of cracks and macroscopic failure patterns of the tunnel supported by different types of rockbolts. **a** PPV = 0.2 m/s; **b** PPV = 0.8 m/s

Figure 17 shows the distribution of cracks and macroscopic failure patterns of the tunnel supported by different types of rockbolts. It is interesting to note that the range of the macroscopic failure zone is the same as that of the tensile damage. This suggests that the initiation, propagation, and development of tensile cracks play a key role in controlling macroscopic failures of surrounding rock masses. The finding agrees that when the stress wave reaches the tunnel surface, the difference in wave impedance between rock and air is excellent. Hence, most of the stress waves are reflected at the surface, causing tensile spalling of the tunnel surrounding rock masses (Wu et al. 2019). As shown in Fig. 17a, when D-bolts are adopted in the tunnel, the extent of rockburst damage is smaller than that of the tunnel supported with Roofex and resin-grouted rebar. Only a few rock blocks are ejected between bolts, and the tunnel surrounding rock masses is overall stable. However, for the tunnel using Roofex and resin-grouted rebar, much more ejected rock blocks are found, and rockfall occurs. The tunnel tend to be unstable. The tunnel tend to be unstable. 

**Fig. 18:**
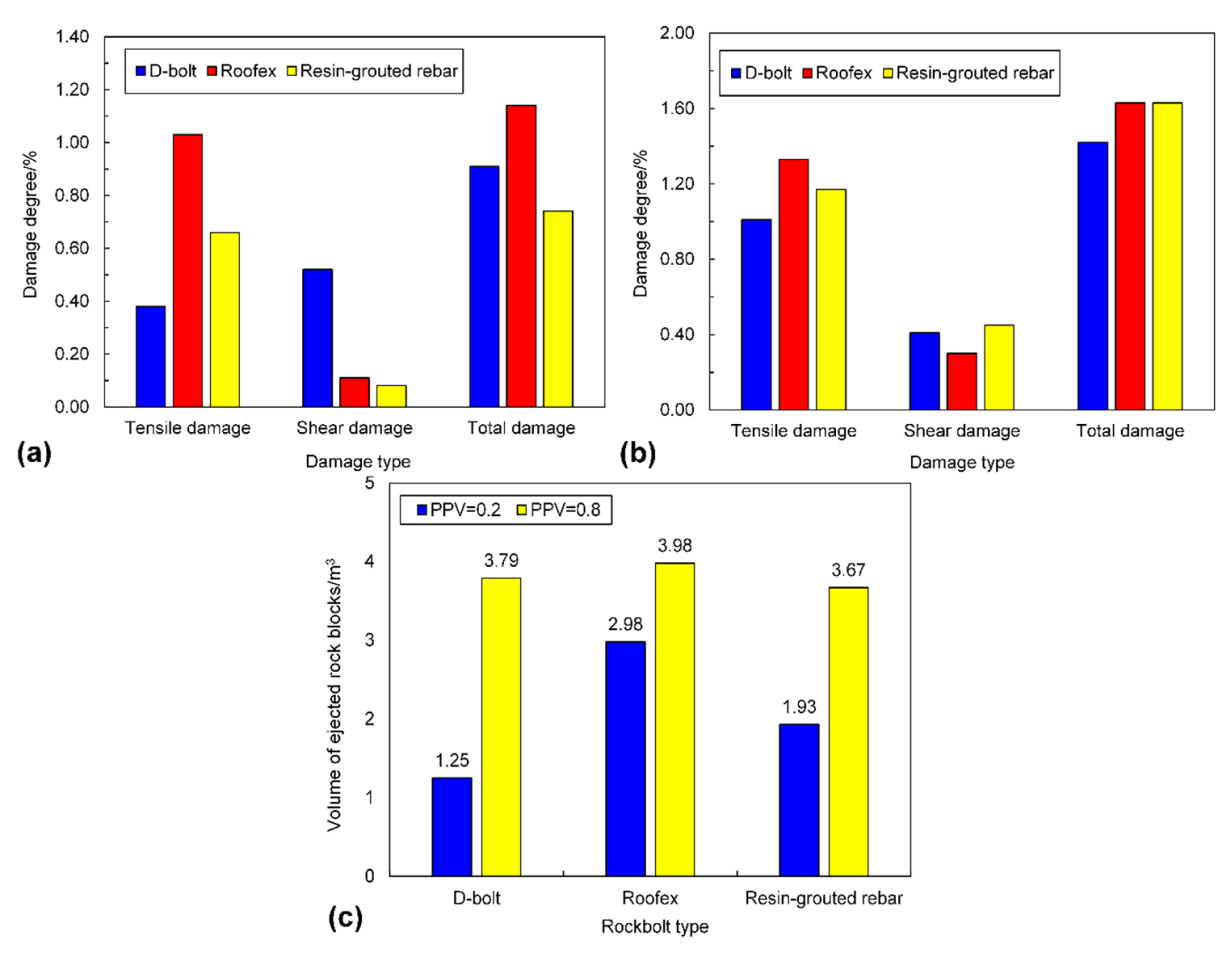
**a** and **b** are the damage degrees of the tunnel when the PPVs are 0.2 and 0.8 m/s, respectively; **c** is the volume of ejected rock blocks of the tunnel induced by rockbursts

The comparison of damage degrees of the tunnel is shown in Fig. 18a, b. It should be noted that the tunnel damage in the excavation stage was excluded since this study focuses on the assessment of the performance of yielding rockbolts during rockbursts. When the PPV is 0.2 m/s (Fig. 18a), the total damage degrees of the tunnel supported by D-bolt and resin-grouted rebar is 0.91% and 0.74%, respectively, which are lower than that of the tunnel using Roofex supporting (1.14%). When the rockburst is violent, the tunnel damage is more serious (Fig. 17b). For example, the total damage degree of the tunnel using D-bolts is increased from 0.91% to 1.42%, with a growth rate of 56% (Fig. 18b). Nevertheless, the minor rockburst damage is found when the tunnel adopts D-bolt supporting, while the total damage degree of the tunnel supported with Roofex and resin-grouted rebar are all 1.63%. It seems that some results are confusing, especially when the PPV is 0.2 m/s. In this scenario, the resin-grouted rebar performs better in mitigating rockbursts damage over D-bolt and Roofex. This is because the resin-grouted rebar is a stiff support fashion. It can effectively restrain the initiation and development of cracks (Yang et al. 2017) and reduce damage degrees. However, when the rockburst is violent, many resin-grouted rebar bolts are broken due to more significant dynamic stresses. The resin-grouted rebar bolts lose their functions to prevent the development of fissures, and therefore the rockburst damage degree is high.

Comparing the volume of ejected rock blocks of the tunnel in three support schemes is shown in Fig. 18c. The volume of ejected rock blocks is the least (1.25 m^3^) when the tunnel uses D-bolt supporting during a weak rockburst (PPV = 0.2 m/s). However, the volume of ejected rock blocks of the tunnel supported with Roofex and resin-grouted rebar is 2.98 and 1.93 m^3^, respectively, which are 2.38 and 1.54 times that of the tunnel supported by D-bolts. The rockburst damage is the most serious when Roofex supports the tunnel due to its lower strength to limit rock deformations and damage. When the PPV is 0.8 m/s, the volume of ejected rock blocks in three support schemes all undergo significant growth. For example, the volume of ejected rock blocks of the tunnel supported by Roofex increases from 2.98 to 3.98 m^3^, with a growth rate of 33.56%. The ejected block volume in this scenario is still the greatest compared to the other two support schemes. The difference between D-bolt and resin-grouted rebar is very little (only 0.12 m^3^). It can also be found that the difference of the ejected block volume between different rockbolt types is similar to that of tunnel deformations and damage degrees, which suggests that the volume of ejected rock blocks is a clear and straightforward variable to evaluate rockburst damage severity.

### Energy evolution analysis

The severity of rockbursts is related to the magnitude of the kinetic energy of ejected rock materials (Cai 2013; Gao et al. 2019). The kinetic energy is one part of the total released energy that the whole supporting system (e.g., rockbolt, cable bolt, liner, and wire mesh) must absorb to reduce rockburst risks (Raffaldi et al. 2017). Therefore, the influences of rockbolt supporting on the distribution and change of kinetic energy were investigated in this study. The kinetic energy of ejected rock blocks was captured by the FISH language programing in UDEC using the following formula:

9 $${W}_{\text{k}}=\sum \frac{1}{2}m{v}^{2}$$

where *m* and *v* are the mass and velocity of ejected rock blocks at the current time step.

**Fig. 19:**
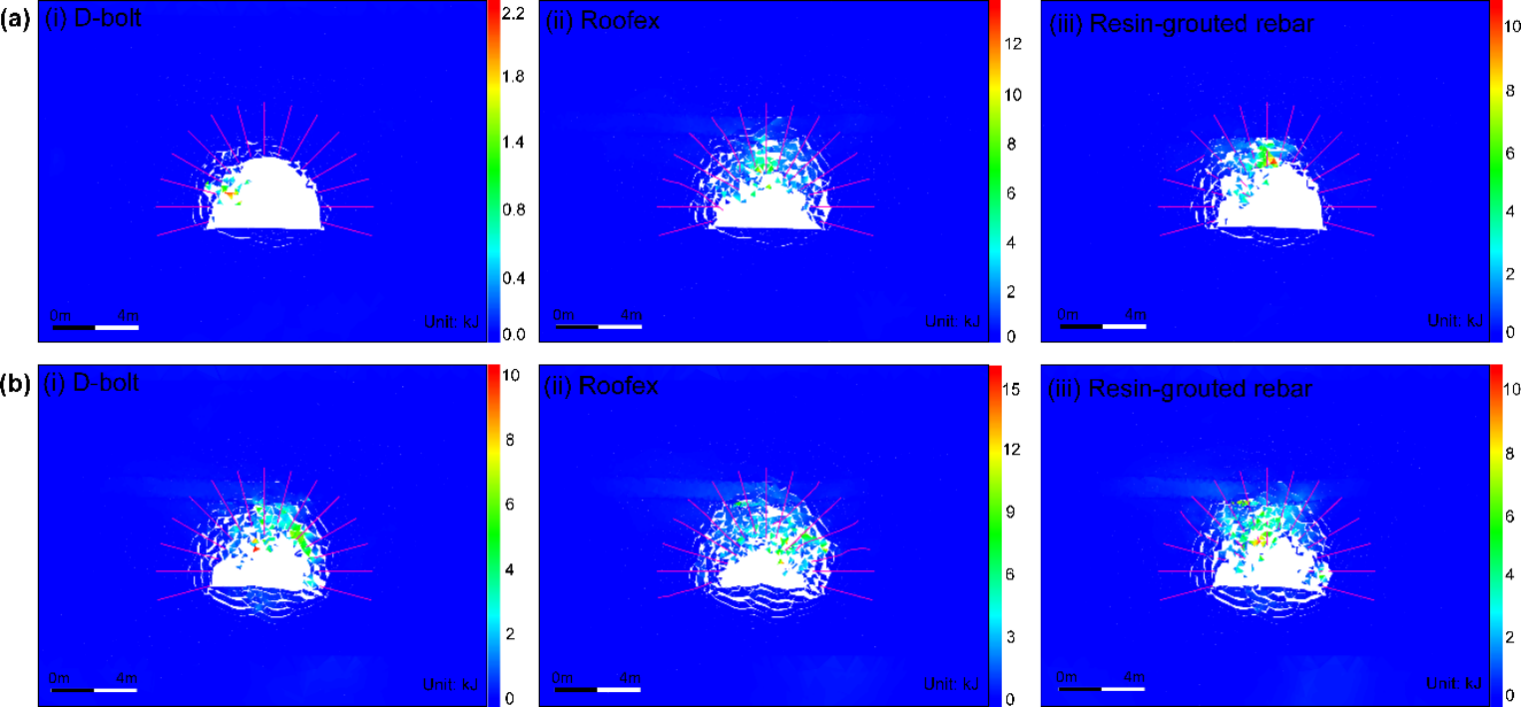
Simulated kinetic energy distribution of ejected rock blocks in the tunnel supported by different types of rockbolts. **a** PPV = 0.2 m/s; **b** PPV = 0.8 m/s

**Fig. 20:**
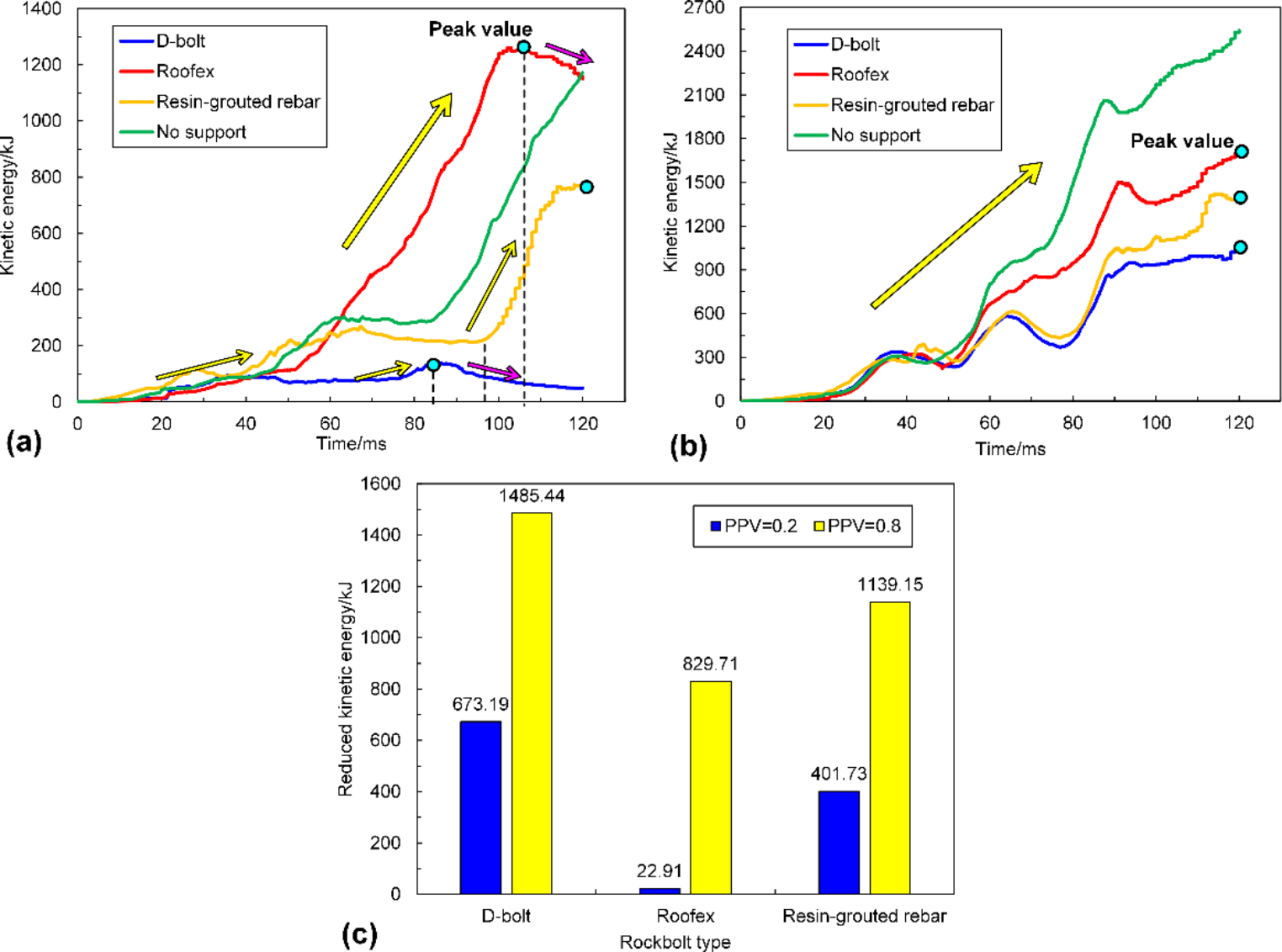
**a** and **b** are the evolution of the kinetic energy of ejected rock blocks in the tunnel when the PPVs are 0.2 and 0.8 m/s, respectively; **c** is the comparison of reduced kinetic energy of ejected rock blocks in the tunnel

The distribution of kinetic energy of ejected rock blocks in three support schemes is shown in Fig. 19. It can be seen that the kinetic energy pattern is very similar to that of velocity (see Fig. 14). As shown in Fig. 19a, only a few rock blocks have relatively high kinetic energy when the D-bolt is adopted. For the tunnel supported by Roofex and resin-grouted rebar, much more rock blocks possess higher kinetic energy. With the increase of PPVs, the range of the region with great velocities grows (see Fig. 14b), and thus more rock blocks possess high kinetic energy (Fig. 19b), which suggests that the rockburst damage is severe. The variation of kinetic energy with time influenced by different rockbolt types is illustrated in Fig. 20a, b. When the PPV is 0.2 m/s, the evolution of kinetic energy in three support schemes can all be divided into two stages. For D-bolt, the kinetic energy first increases slowly to the peak value from 0 to 84 ms and then gradually declines. For Roofex, the kinetic energy experiences fast growth, especially after 52 ms, and reaches the peak value at 105 ms. Then, the kinetic energy drops with time but is still at a high level. When the tunnel is supported by resin-grouted rebar, the kinetic energy first increases slowly to a plateau from 0 to 97 ms and then suffers a sudden surge.

In contrast, the kinetic energy of ejected rock blocks in three support schemes all increase almost linearly with time when the PPV is 0.8 m/s, although they may experience several fluctuations. In summary, when the PPV is 0.2 m/s, D-bolts absorb the kinetic energy of ejected rock blocks effectively, and the rockburst is controlled. However, they cannot absorb sufficient kinetic energy to control a violent rockburst successfully. Roofex and resin-grouted rebar fail to reduce the kinetic energy of ejected rock blocks effectively and cannot solely control weak and strong rockbursts.

To further evaluate the dynamic energy-absorption capacity of three types of rockbolts, the tunnel without adopting any supports during rockbursts (PPVs are 0.2 and 0.8 m/s) was simulated. Then, a new variable was defined as the reduced kinetic energy, which is the difference between the final kinetic energy of ejected rock blocks in the tunnel without and using rockbolts. It should be noted that the calculation of the energy-absorption magnitude of rockbolts is not feasible because the action of rockbolts during rockbursts is very complex and is no longer a simple pull-out test or drop test (Zhang and Nordlund 2019). Thus, the indirect calculation method used in this study can be accepted as a relatively rational estimation to assess the dynamic energy-absorption capacity of rockbolts. Figure 20c compares reduced kinetic energy of ejected rock blocks in the tunnel supported by different rockbolts. The reduced kinetic energy is the highest (673.19 and 1485.44 kJ for PPV = 0.2 and 0.8 m/s) when the tunnel uses D-bolt supporting. By comparison, the reduced kinetic energy is the lowest (22.91 and 829.71 kJ) for the tunnel supported by Roofex, while the performance of resin-grouted rebar on reducing kinetic energy is in between the D-bolt and Roofex. It might be argued that the Roofex as a type of yielding rockbolt, having a higher energy-absorption capacity than resin-grouted rebar (20.94 vs. 4.15 kJ, see Table 3), should reduce more kinetic energy than resin-grouted rebar. This could be because Roofex has a lower strength, and its sliding mechanism can be easily activated. Thus, it is too “soft” or “smooth” to limit ejected rocks’ movement compared to the resin-grouted rebar and D-bolts. It can also be seen that the reduced kinetic energy grows with the increasing PPVs. This law is consistent with some published simulation results (Raffaldi et al. 2017; Raffaldi and Loken 2016). Therefore, it can be concluded that the dynamic energy-absorption capacity of rockbolts is affected by rockburst magnitudes, which should be considered in rockburst support designs. This finding also verifies the hypothesis that the performance of rockbolts during rockbursts is complex, while the dynamic energy-absorption capacity of rockbolts obtained from drop tests is usually a constant value (Bosman et al. 2018).

### Rockbolt force analysis

**Fig. 21:**
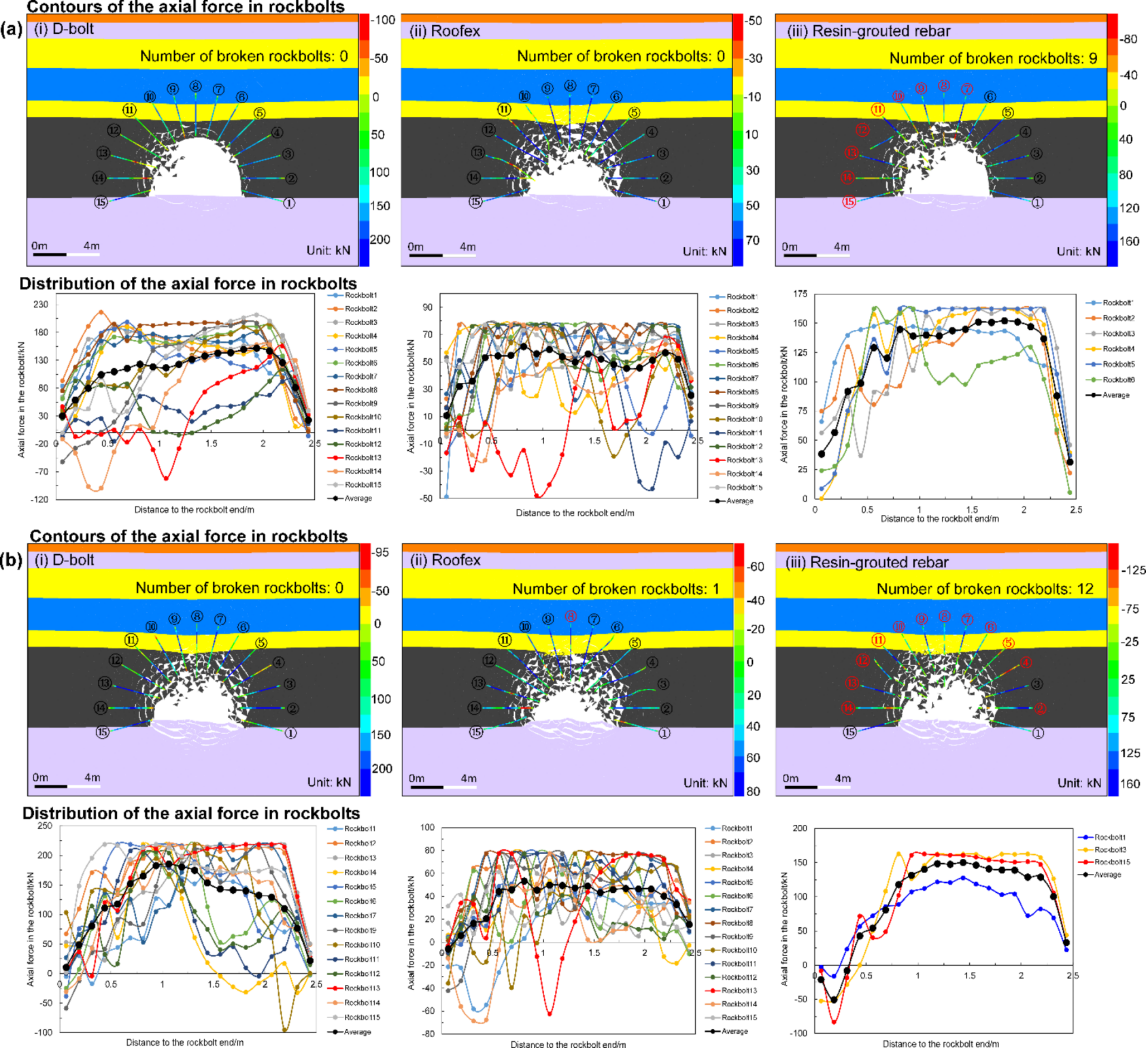
Simulated contours and distribution of the axial force in rockbolts for the tunnel supported by different rockbolts. **a** PPV = 0.2 m/s; **b** PPV = 0.8 m/s. The black and red numbers indicate intact and broken rockbolts, respectively. The positive value of axial forces represents a tensile load

The simulated axial force distribution of rockbolts in three support schemes is shown in Fig. 21. It can be seen that in all three cases, the tensile axial force tends to reach the peak value at a certain distance (around 1.0–1.5 m) from the bolt end (head) and then gradually decreases to a low value. The simulated axial force patterns of rockbolts agree with some published experimental (Hyett et al. 1996) and numerical simulation results (Lisjak et al. 2020; Ma et al. 2014). When the PPV is 0.2 m/s (Fig. 21a), the average peak values of axial forces for three rockbolt types are 151.77, 61.27, and 151.05 kN, respectively. Thus, both the D-bolt and resin-grouted rebar can bear the high load of rock masses, while the Roofex cannot provide sufficient resistance to control large rock deformation and rapid rock bulking during rockbursts.

**Fig. 22:**
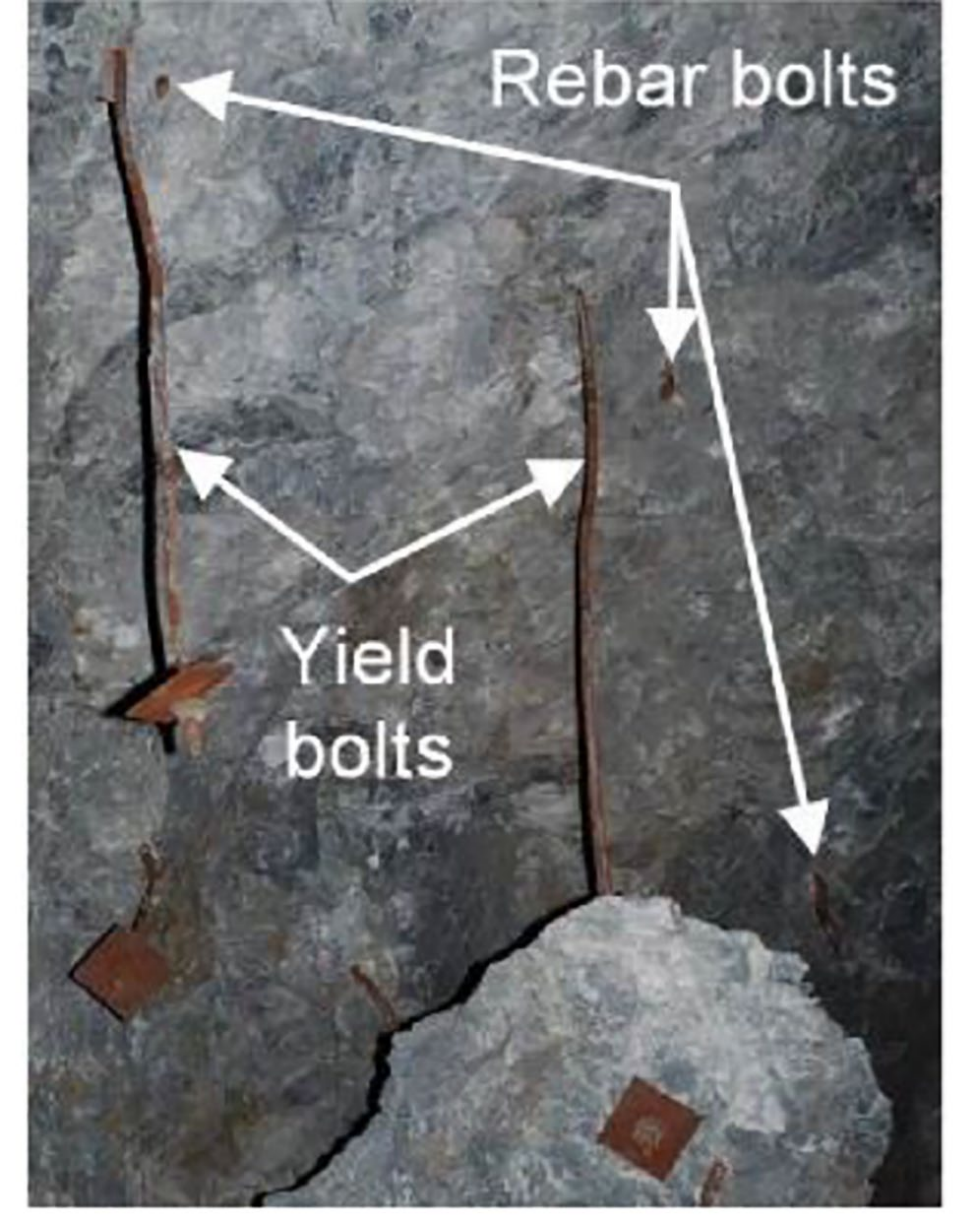
Observed performance of fully resin-grouted rebar and yielding rockbolts in a rockburst (Li 2021)

Additionally, it can be observed that nine resin-grouted rebar bolts are broken, resulting in the unsuccessful control of the rockburst. Again, this is because the resin-grouted rebar has limited deformation capacity to accommodate rapid rock bulking and relieve rock ejection (Cai 2013; Kaiser and Cai 2012). There are no broken rockbolts found for the tunnel adopting D-bolt and Roofex supporting. This finding agrees well with many in situ observations (Cai et al. 2010, 2019; Charette and Plouffe 2007; Li 2021). An example is shown in Fig. 22. When the PPV is 0.8 m/s (Fig. 21b), the simulated axial force patterns of rockbolts resemble a weak rockburst (PPV = 0.2 m/s). The average peak values of axial forces for three rockbolt types are 185.49, 53.30 kN, and 151.17 kN, respectively. However, more rockbolts are broken due to violent rock ejection and bulking. No. 9 D-bolt in the middle roof is broken, while 12 resin-grouted rebar bolts are found to be broken in the roof and two sidewalls, with an increase of three broken bolts.

Furthermore, it can also be observed that the location of broken rockbolts mainly depends on the rock ejection direction (Fig. 21a (iii), b (iii)). In summary, the D-bolt and resin-grouted rebar can maintain a high axial force level during rockbursts to restrain rock ejection and bulking, but the resin-grouted rebar is prone to be broken due to a minimal elongate rate failing to mitigate rockburst damage effectively. Roofex’s axial force is too low to control rockburst, although it has an excellent deformation capacity over the other two rockbolt types. Besides, the axial force patterns and the intactness of D-bolt and Roofex verify the reliability and rationality of the “rockbolt” element in modeling the performance of yielding rockbolts.

## Discussion

### Effects of floor supporting

As mentioned above, significant floor heaving occurs in violent rockbursts according to current simulation results and in situ observations (Mutke et al. 2009; Prusek and Masny 2015). Besides, the rockbolt supporting in roof and sidewalls do not have notable influences on the floor response. Therefore, it is interesting to explore whether yielding rockbolts can be used to restrain floor heaving induced by rockbursts or not. Since the D-bolt performs better on controlling rockbursts than Roofex and resin-grouted rebar based on previous analyses, it was decided to simulate the tunnel supported by D-bolts with floor supporting during a violent rockburst (PPV = 0.8 m/s). The floor is supported by five D-bolts with a length of 2.5 m and row spacing of 1.4 m, while other parameters remain constant.

**Fig. 23:**
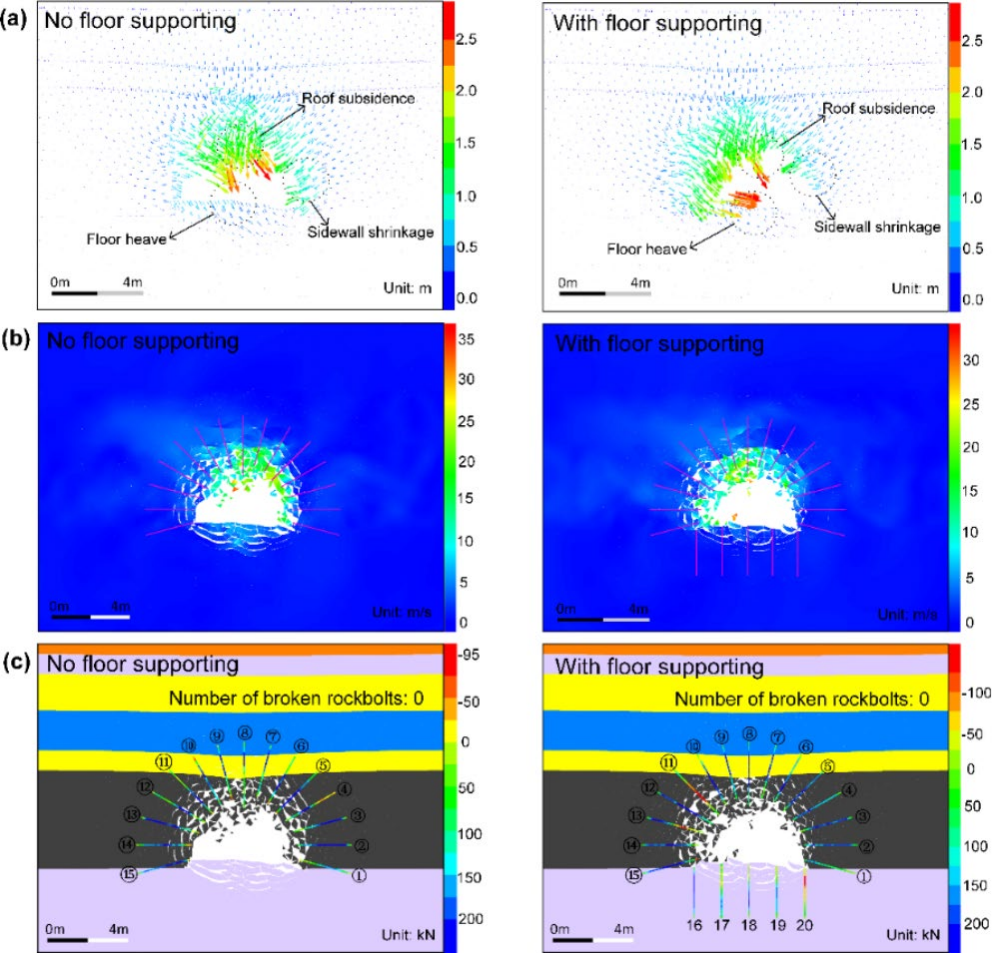
**a**, **b** and **c** are simulated displacement vectors, velocity distribution, and macroscopic failure patterns of the tunnel and rockbolt axial forces

**Fig. 24:**
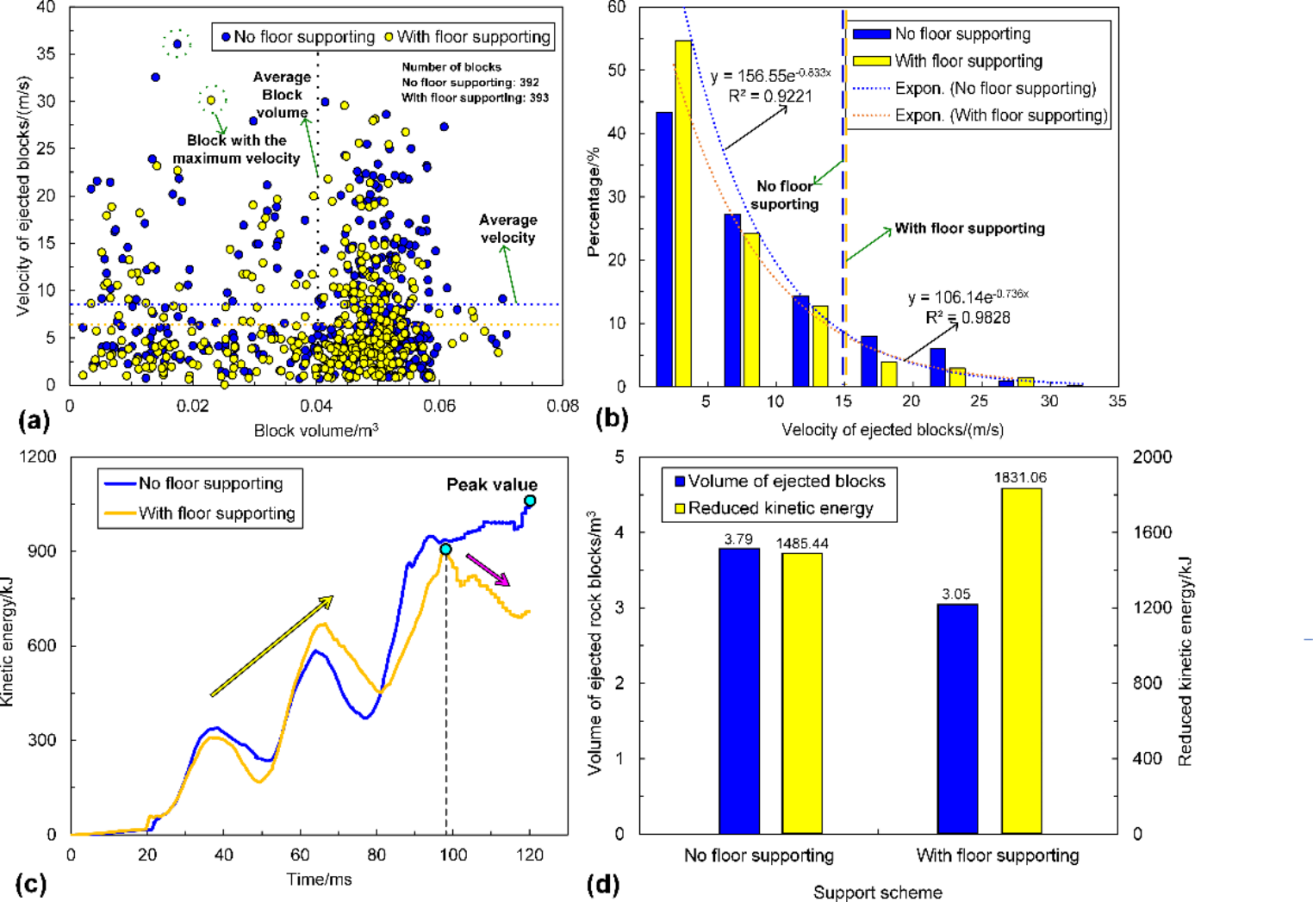
**a** is the velocity of all detached blocks versus block volume; **b** is the velocity distribution of all detached blocks; **c** is the evolution of the kinetic energy of ejected rock blocks in the tunnel; **d** is the comparison of the volume of ejected rock blocks and reduced kinetic energy

The simulation results are shown in Figs. 23 and 24. It can be seen from Fig. 23a that the floor heaving is reduced, although it still occurs when the tunnel adopts floor supporting. The floor heaving value is dropped from 320.5 to 148.5 mm, with a decrease rate of 53.67%. As shown in Fig. 23b, the velocities of rock blocks in the floor are significantly reduced, which are lower than 3 m/s. By comparison, the velocities can be up to 5–8 m/s when the floor is not supported. The statistical analysis results of the velocities of all detached rock blocks are illustrated in Fig. 24a, b. As shown in Fig. 24a, the average velocity of rock blocks in the tunnel without floor supporting is 8.34 m/s, while the average velocity decreases by 1.64 m/s to 6.70 m/s when the floor is supported. In addition, the velocity distributions of rock blocks in these two scenarios are all extensive.

Figure 24b shows that 84.8% of rock blocks in the tunnel with floor supporting possesses a velocity lower than 15 m/s, while 91.7% of rock blocks in the tunnel without floor supporting are within the same velocity range. Figure 23c also shows that the severity of floor heaving is reduced, but rockfall and rock ejection are still observed. The variation of kinetic energy with time in two scenarios is illustrated in Fig. 24c. The kinetic energy of ejected rock blocks almost increases linearly when the floor is not supported, although it may experience several fluctuations. In contrast, kinetic energy evolution can be divided into two stages when the floor using D-bolts. The kinetic energy first increases to the peak value from 0 to 99 ms and then gradually declines. This is because more rockbolts are deformed to absorb the kinetic energy of ejected rock blocks, which the lower average velocity can also confirm.

Additionally, Fig. 24d also shows that the tunnel suffers lower damage when using floor supporting. The volume of ejected rock blocks is decreased by 0.74 m^3^ to 3.05 m^3^, while the reduced kinetic energy increases to 1831.06 kJ, with a growth rate of 23.27%. These results suggest that floor heaving and the tunnel’s rockburst damage can be mitigated by floor supporting with D-bolts as the tunnel surrounding rock is an integrity system (Wang et al. 2018). However, rock blocks’ average velocity and kinetic energy are still high, indicating that the new support scheme fails to control a strong rockburst.

### Effects of cable bolts

Previous analyses show that supporting three types of rockbolts solely are unable to control violent rockbursts. This is because the effective support length of some rockbolts is less than the depth of the rock loose circle resulting in the instability of the tunnel (Wang et al. 2015). Therefore, it is exciting and necessary to discuss the possibility of strengthening the support system, e.g., using cable bolts. The application of cable bolts is common in many burst-prone underground tunnels (Cai 2013). The cable bolts with high strengths and pre-stress can restrain the initiation and development of tensile and shear cracks and resist the large deformation, thereby strengthening surrounding rock masses and maintaining their integrity (reinforcement function). Cable bolts usually have a great length, holding retaining elements and overhanging the combined arch of bolt supporting back to stable areas in depth (excavation influenced zone, Wang et al. 2015). Besides, the cable bolts also have a relatively high elongation rate (4%–7%, Kang et al. 2009) to absorb the deformation energy induced by rock bulking. Hence, the tunnel supported by D-bolts plus seven plain cable bolts during a violent rockburst (PPV = 0.8 m/s) was simulated in this research. The plain cable bolts have a length of 7.2 m and row spacing of 1.7 m, while other parameters remain constant. The input parameters of the cable element in UDEC are adopted from Chen et al. (2016), Gao et al. (2015), and Yang et al. (2017), as listed in Table 7.

**Table 7:** Input parameters of the cable element

Input parameters	Cross-sectional area (m^2^)	Density (kg/m^3^)	Elastic modulus (GPa)	Tensile yield strength (kN)	Grout stiffness (GN/m/m)	Grout strength (kN)
Cable	3.14 × 10^− 4^	7500	200	500	2	400

**Fig. 25:**
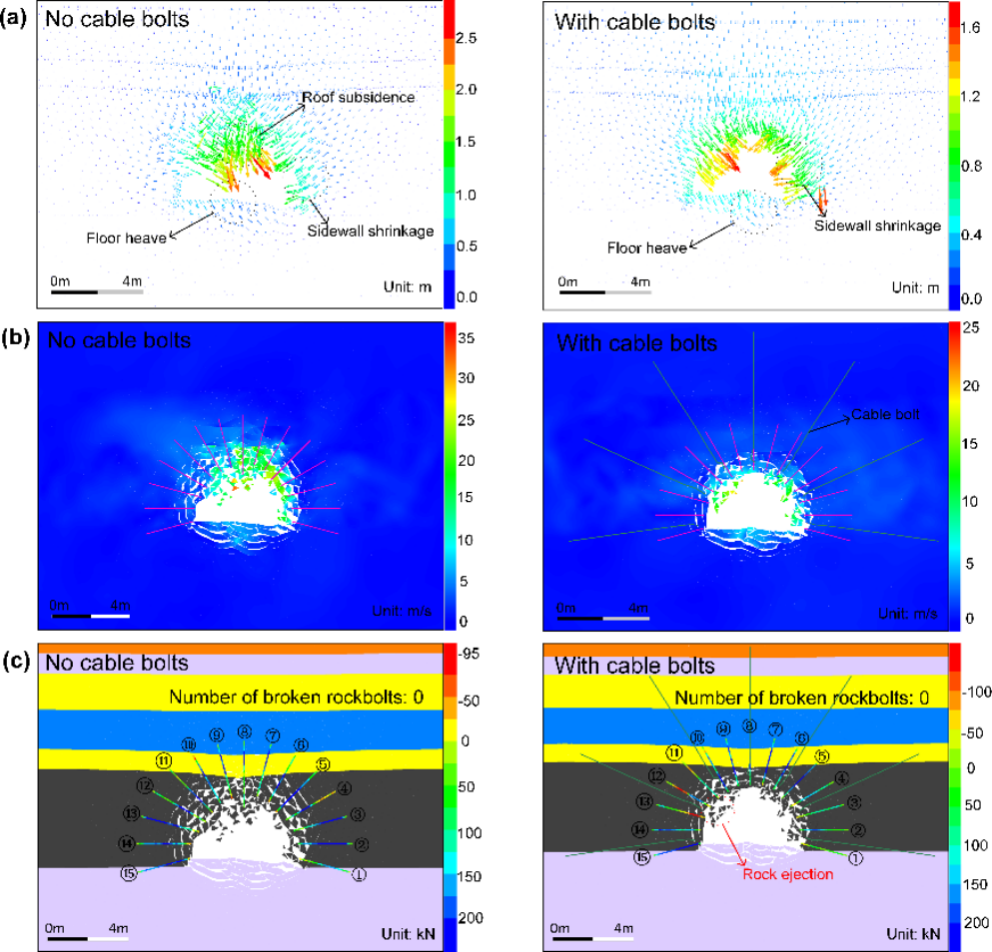
**a**, **b** and **c** are simulated displacement vectors, velocity distribution, and macroscopic failure patterns of the tunnel and rockbolt axial forces

**Fig. 26:**
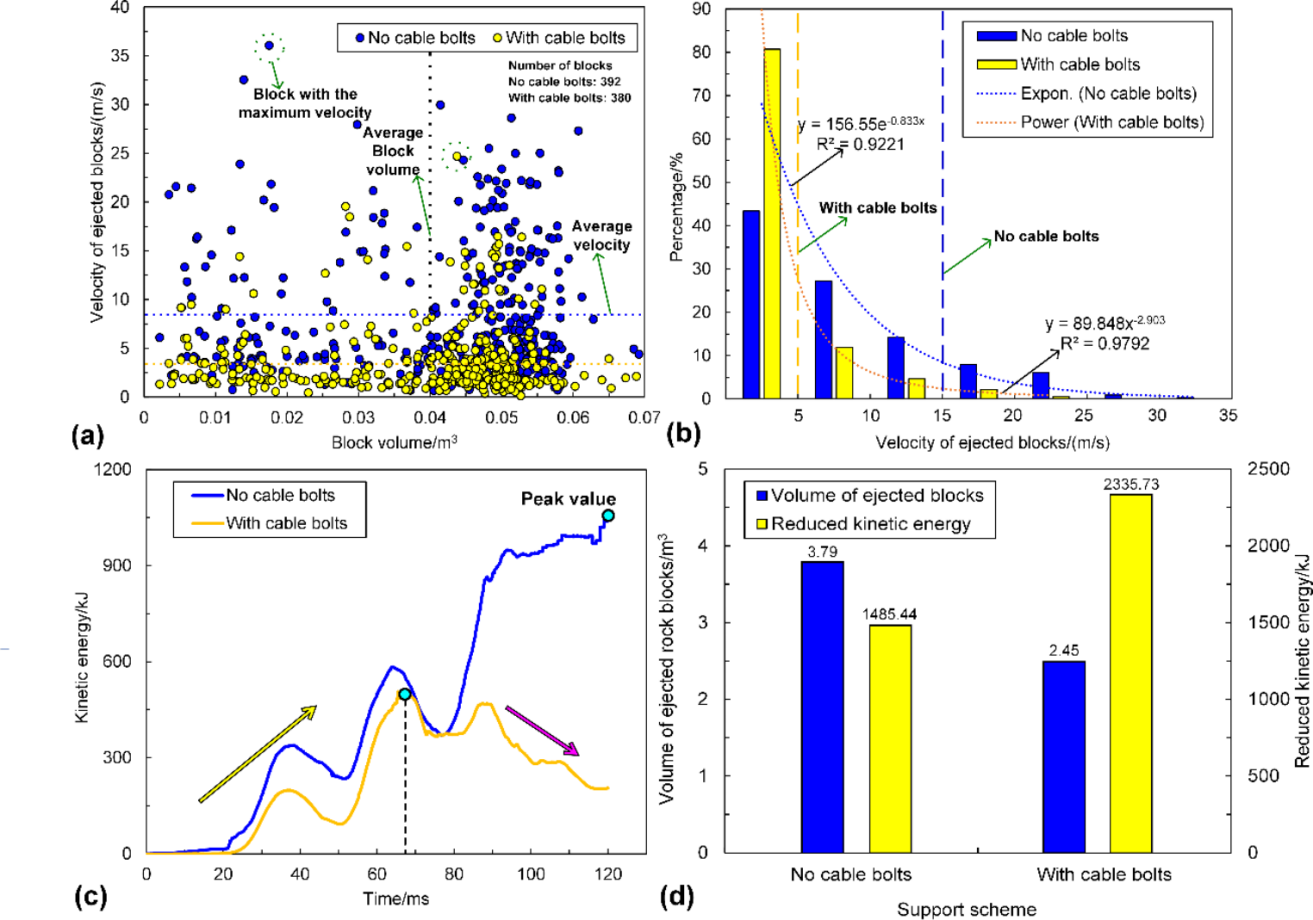
**a** is the velocity of all detached blocks versus block volume; **b** is the velocity distribution of all detached blocks; **c** is the evolution of the kinetic energy of ejected rock blocks in the tunnel; **d** is the comparison of the volume of ejected rock blocks and reduced kinetic energy

The simulation results are shown in Figs. 25 and 26. Figure 25a shows that the roof subsidence is reduced while sidewall shrinkage and floor heaving still occur when the tunnel adopts cable bolts. The roof subsidence magnitude is dropped from 1459.3 to 711.1 mm, with a decrease rate of 51.27%. As shown in Fig. 25b, the range of the region with high velocities is significantly reduced, and fewer ejected rock blocks are observed compared to the tunnel without cable supporting. The statistical analysis results of the velocities of all detached rock blocks are illustrated in Fig. 26a, b. As shown in Fig. 26a, the average velocity of rock blocks is decreased from 8.34 to 3.61 m/s when using cable bolts, with a decrease rate of 56.71%. Besides, the velocity distributions of rock blocks in the tunnel using cable bolts are less extensive. Figure 26b shows that 84.8% of rock blocks in the tunnel without cable supporting possesses the velocity lower than 15 m/s, while 80.72% of rock blocks are within the velocity range of 0–5 m/s for the tunnel using cable bolts. Figure 25c depicts that the severity of rockburst damage is remarkably reduced because the rock ejection only occurs in several local areas between rockbolts. This is mainly due to the absence of surface retaining elements (e.g., shotcrete and wire mesh). The variation of kinetic energy with time in two scenarios is illustrated in Fig. 26c. Like the tunnel using floor supporting, kinetic energy evolution can be divided into two stages when the tunnel adopts cable bolts. The kinetic energy first increases to the peak value from 0 to 67 ms and then gradually declines and reaches a lower level. This indicates that cable bolts absorb much kinetic energy and can effectively restrain the detachment and ejection of rock blocks. A much lower average velocity can also confirm the finding.

Additionally, Fig. 26d shows that the tunnel suffers lower damage when using cable supporting. The volume of ejected rock blocks is decreased by 1.34 m^3^ to 2.45 m^3^, while the reduced kinetic energy increases to 2335.73 kJ, with a growth rate of 57.24%. These results suggest that the rockburst damage severity can be significantly reduced by additional support with cable bolts. It could also be anticipated that the new support scenario can control violent rockbursts if rational surface retaining elements are applied.

## Conclusions

In this paper, the performance of yielding rockbolts during rockbursts with actual seismic loading are thoroughly evaluated using numerical modeling method. Two types of yielding rockbolts (D-bolt and Roofex) and the fully resin-grouted rebar bolt are modeled via the “rockbolt” element in UDEC. A 2D model of a deep tunnel is built to evaluate the performance (e.g., the dynamic capacity of energy-absorption and control of rock damage) of yielding and traditional rockbolts based on the simulated rockbursts. The influence of different rockburst magnitudes is also studied. The main conclusions are as follows:

1. The “rockbolt” element in UDEC can be used to simulate the performance of yielding rockbolts. The volume of ejected rock blocks is a clear and straightforward variable to evaluate rockburst damage severity. The reduced kinetic energy can be accepted as a relatively rational estimation to assess the dynamic energy-absorption capacity of rockbolts.
2. The D-bolt can effectively control and mitigate rockburst damage during a weak rockburst because of its high strength and deformation capacity. The Roofex is too “soft” or “smooth” to limit ejected rocks’ movement and restrain the large deformation induced by rockbursts, although it has an excellent deformation capacity. The resin-grouted rebar bolt can main a high axial force level during rockbursts, but its elongation rate is very low and is easy to break during dynamic shocks, which cannot control rapid rock bulking or ejection effectively. These two types of rockbolts fail to control a weak rockburst.
3. The rockburst is more severe when the PPV is higher. Three types of rockbolts solely fail to control the large deformation and mitigate rockburst damage during violent rockbursts. Nevertheless, the D-bolt still has a better performance in controlling rockbursts over the other two rockbolt types. Additional measures, e.g., using cable bolts and steel arch, and/or distress drilling and blasting methods should be supplemented to control violent rockbursts.
4. The floor heaving and the rockburst damage of the whole tunnel can be mitigated by floor supporting with D-bolts. The rockburst damage severity can be significantly reduced by additional support with cable bolts. It could be anticipated that supporting with high strength yielding rockbolts and cable bolts can control violent rockbursts if rational surface retaining elements are applied.

This study highlights the effectiveness of numerical modeling methods in assessing the complex performance of yielding rockbolts during rockbursts, which can provide some references to improve and optimize the design of rock supporting in burst-prone grounds. However, further research work can be done in the following aspects:

1. Only the simplified P wave was used in this study. However, the actual dynamic disturbance is composed of P and S waves and is usually very complex. The performance of yielding rockbolts should be further examined if actual seismic data are available.
2. The performance of yielding rockbolts was mainly evaluated from the “macro” views of the dynamic energy-absorption capacity and the control of the deformation and damage of rock masses. Other “micro” behavior of rockbolts, e.g., the shear force and failure of rockbolt-grout interfaces, will be studied in future research.
3. The surface retaining element (e.g., fiber-reinforced shotcrete, wire mesh, and steel arch) is an indispensable component of the support system as it can prevent the unraveling of fractured rocks between rockbolts. Therefore, the effects of the combination of surface retaining elements, yielding rockbolts, and cable bolts on controlling rockbursts should be further investigated.

## References

[CR1] Atlas Copco Construction Mining Technique (2009) Roofex rock bolt. https://www.forconstructionpros.com/equipment/underground/vertical-drilling-equipment/product/10080325/atlas-copco-construction-mining-technique-usa-llc-roofex-rock-bolt. Accessed 22 May 2021

[CR2] Bahrani N, Hadjigeorgiou J (2017). Explicit reinforcement models for fully-grouted rebar rock bolts. J Rock Mech Geotech.

[CR3] Bennett TJ, Marshall ME (2001) Identification of rockbursts and other mining events using regional signals at international monitoring system stations. SCIENCE APPLICATIONS INTERNATIONAL CORP MCLEAN VA

[CR4] Blake W(1972) Rock-burst mechanics.J Q Colo Sch Mines67(1)

[CR5] Blake W, Hedley DG (2003). Rockbursts: case studies from north american hard-rock mines.

[CR6] Bosman K, Cawood M, Berghorst A(2018) Relationship between energy per impulse and dynamic capacity of a rockbolt. In: Proceedings of Rock Dynamics–Experiments, Theories and Applications. CRC Press, Trondholm, Norway, pp 379–384

[CR7] Cai M (2013). Principles of rock support in burst-prone ground. Tunn Undergr Space Technol.

[CR8] Cai M, Champaigne D, Kaiser PK(2010) Development of a fully debonded cone bolt for rockburst support. In: Proceedings of the Fifth International Seminar on Deep and High Stress Mining. Australian Centre for Geomechanics, Santiago, pp 329–342

[CR9] Cai M, Champaigne D, Coulombe JG, Challagulla K (2019). Development of two new rockbolts for safe and rapid tunneling in burst-prone ground. Tunn Undergr Space Technol.

[CR10] Charette F, Plouffe M(2007) Roofex®–Results of laboratory testing of a new concept of yieldable tendon. In: Proceedings of the Fourth International Seminar on Deep and High Stress Mining. Australian Centre for Geomechanics, Perth, Australia, pp 395–404

[CR11] Chen M, Yang SQ, Zhang YC, Zang CW (2016). Analysis of the failure mechanism and support technology for the Dongtan deep coal roadway. Geo Eng.

[CR12] Dai L, Pan Y, Li Z, Wang A, Xiao Y, Liu F, Shi T, Zheng W (2021). Quantitative mechanism of roadway rockbursts in deep extra-thick coal seams: Theory and case histories. Tunn Undergr Space Technol.

[CR13] Diederichs MS (2018). Early assessment of dynamic rupture hazard for rockburst risk management in deep tunnel projects. J South Afr Inst Min Metall.

[CR14] Dammyr Ø (2016). Prediction of Brittle Failure for TBM Tunnels in Anisotropic Rock: A Case Study from Northern Norway. Rock Mech Rock Eng.

[CR15] Fairhurst C, Hudson JA (1999). Draft ISRM suggested method for the complete stress-strain curve for intact rock in uniaxial compression. Int J Rock Mech Min Sci.

[CR16] Farrokh E, Rostami J (2009). Effect of adverse geological condition on TBM operation in Ghomroud tunnel conveyance project. Tunn Undergr Space Technol.

[CR17] Gao F, Kaiser PK, Stead D, Eberhardt E, Elmo D (2019). Strainburst phenomena and numerical simulation of self-initiated brittle rock failure. Int J Rock Mech Min Sci.

[CR18] Gao F, Kang H, Li J (2021) Numerical simulation of fault-slip rockbursts using the distinct element method. Tunn Undergr Space Technol 110. 10.1016/j.tust.2020.103805

[CR19] Gao F, Stead D, Kang H (2015). Numerical simulation of squeezing failure in a coal mine roadway due to mining-induced stresses. Rock Mech Rock Eng.

[CR20] Ghorbani M, Shahriar K, Sharifzadeh M, Masoudi R (2020). A critical review on the developments of rock support systems in high stress ground conditions. Int J Min Sci Technol.

[CR21] He M, Xia H, Jia X, Gong W, Zhao F, Liang K (2012). Studies on classification, criteria and control of rockbursts. J Rock Mech Geotech.

[CR22] Hoek E, Carranza-Torres C, Corkum B(2002) Hoek-Brown failure criterion-2002 edition. In: Proceedings of NARMS-Tac. 267–273

[CR23] Hoek E, Diederichs M (2006). Empirical estimation of rock mass modulus. Int J Rock Mech Min Sci.

[CR24] Hu L, Su G, Liang X, Li Y, Yan L (2020) A distinct element based two-stage-structural model for investigation of the development process and failure mechanism of strainburst. Comput Geotech 118. 10.1016/j.compgeo.2019.103333

[CR25] Hyett A, Moosavi M, Bawden W (1996). Load distribution along fully grouted bolts, with emphasis on cable bolt reinforcement. Int J Numer Anal Methods Geomech.

[CR26] Itasca (2020). UDEC Version 7.0 Manual.

[CR27] Kaiser PK, Cai M (2012). Design of rock support system under rockburst condition. J Rock Mech Geotech.

[CR28] Kaiser PK, McCreath D, Tannant D (1996) Canadian rockburst support handbook. Geomechanics Research Center

[CR29] Kang H, Lin J, Wu Y(2009) High pretensioned stress and intensive cable bolting technology set in full section and application in entry affected by dynamic pressure.J Chin Coal Soci9 (in Chinese)

[CR30] Kong P, Jiang L, Jiang J, Wu Y, Chen L, Ning J (2019) Numerical analysis of roadway rock-burst hazard under superposed dynamic and static loads. Energies 12(19). 10.3390/en12193761

[CR32] Li CC (2021). Principles and methods of rock support for rockburst control. J Rock Mech Geotech.

[CR33] Li CC, Doucet C (2011). Performance of D-bolts under dynamic loading. Rock Mech Rock Eng.

[CR34] Li CC, Stjern G, Myrvang A (2014). A review on the performance of conventional and energy-absorbing rockbolts. J Rock Mech Geotech.

[CR35] Lisjak A, Young-Schultz T, Li B, He L, Tatone BSA, Mahabadi OK (2020) A novel rockbolt formulation for a GPU-accelerated, finite-discrete element method code and its application to underground excavations. Int J Rock Mech Min Sci 134. 10.1016/j.ijrmms.2020.104410

[CR36] Liu X(2017) Study on the mechanism and control of rockburst in roadways under dynamic loads. Dissertation, Shandong University of Science and Technology

[CR37] Lysmer J, Kuhlemeyer RL (1969). Finite dynamic model for infinite media. J Eng Mech Div.

[CR38] Ma S, Nemcik J, Aziz N (2014). Simulation of fully grouted rockbolts in underground roadways using FLAC2D. Can Geotech J.

[CR39] Małkowski P, Ostrowski Ł(2019) Convergence monitoring as a basis for numerical analysis of changes of rock-mass quality and Hoek-Brown failure criterion parameters due to longwall excavation.Arch Min93–118

[CR40] Małkowski P, Ostrowski Ł, Bachanek P (2017). Modelling the small throw fault effect on the stability of a mining roadway and its verification by in situ investigation. Energies.

[CR41] Manouchehrian SMA(2016) Numerical Modeling of Unstable Rock Failure. Dissertation, Laurentian University

[CR42] Marambio E, Vallejos J, Burgos L, Gonzalez C, Castro L, Saure J, Urzua J(2018) Numerical modelling of dynamic testing for rock reinforcement used in underground excavations. In: Proceedings of the Fourth International Symposium on Block and Sublevel Caving. Australian Centre for Geomechanics, pp 767–780

[CR43] Marinos P, Hoek E(2000) GSI: a geologically friendly tool for rock mass strength estimation. In: Proceedings ISRM international symposium. International Society for Rock Mechanics and Rock Engineering

[CR44] Mitri H (2000) Practitioner’s guide to destress blasting in hard rock mines. McGill University

[CR45] Mortazavi A, Tabatabaei Alavi F (2013). A numerical study of the behavior of fully grouted rockbolts under dynamic loading. Soil Dyn Earthq Eng.

[CR46] Mutke G (2016) Peak particle velocity as an indicator of dynamic load exerted on the support of underground workings. Acta Geodyn Geomater 367–378. 10.13168/agg.2016.0019

[CR47] Mutke G, Dubiński J, Lurka A (2015). New criteria to assess seismic and rock burst hazard in coal mines. Arch Min.

[CR48] Mutke G, Lurka A, Dubiński J(2009) Seismic monitoring and rock burst hazard assessment in Deep Polish Coal Mines–Case study of rock burst on April 16, 2008 in Wujek-Slask Coal Mine. In: 7th International Symposium on Rockburst and Seismicity in Mines (RASiM 7): Controlling Seismic Hazard and Sustainable Development of Deep Mines. CA Tang (ed.). Rinton Press, pp 1413–1424

[CR49] Naji A, Rehman H, Emad M, Yoo H (2018) Impact of shear zone on rockburst in the deep neelum-jehlum hydropower tunnel: a numerical modeling approach. Energies 11(8). 10.3390/en11081935

[CR50] Nie W, Zhao ZY, Ning YJ, Guo W (2014). Numerical studies on rockbolts mechanism using 2D discontinuous deformation analysis. Tunn Undergr Space Technol.

[CR51] Normet(2021) Normet D-Bolt® – Dynamic rock bolt. https://www.normet.com/product/d-bolt/. Accessed 22 May 2021

[CR52] Ortlepp W(1992) The design of support for the containment of rockburst damage in tunnels: an engineering approach. In: Proceedings International symposium on rock support. pp 593–609

[CR53] Prusek S, Masny W (2015). Analysis of damage to underground workings and their supports caused by dynamic phenomena. J Min Sci.

[CR54] Pu Y, Apel DB, Liu V, Mitri H (2019). Machine learning methods for rockburst prediction-state-of-the-art review. Int J Min Sci Technol.

[CR55] Raffaldi M, Chambers D, Johnson J(2017) Numerical study of the relationship between seismic wave parameters and remotely triggered rockburst damage in hard rock tunnels. In: Proceedings of the Eighth International Conference on Deep and High Stress Mining. Australian Centre for Geomechanics, pp 373–386

[CR56] Raffaldi M, Loken M(2016) Framework for simulating fracture, ejection, and restraint of rock around a mine drift subjected to seismic loading. In: 50th US Rock Mechanics/Geomechanics Symposium. American Rock Mechanics Association

[CR57] Sharifzadeh M, Lou J, Crompton B (2020). Dynamic performance of energy-absorbing rockbolts based on laboratory test results. Part I: Evolution, deformation mechanisms, dynamic performance and classification Tunn. Undergr Space Technol.

[CR58] Skrzypkowski K (2021) An experimental investigation into the stress-strain characteristic under static and quasi-static loading for partially embedded rock bolts. Energies 14(5). 10.3390/en14051483

[CR59] Skrzypkowski K, Korzeniowski W, Zagórski K, Zagórska A (2020) Modified rock bolt support for mining method with controlled roof bending. Energies 13(8). 10.3390/en13081868

[CR60] Stavrou A, Vazaios I, Murphy W, Vlachopoulos N (2019). Refined approaches for estimating the strength of rock blocks. Geotech Geol Eng.

[CR61] Stillborg B (1994). Professional Users Handbook for Rock Bolting.

[CR62] Stjern G(1995) Practical performance of rock bolts. Dissertation, University of Trondheim

[CR63] Szott W, Słota-Valim M, Gołąbek A, Sowiżdżał K, Łętkowski P (2018). Numerical studies of improved methane drainage technologies by stimulating coal seams in multi-seam mining layouts. Int J Rock Mech Min Sci.

[CR64] Tan X, Konietzky H (2014). Brazilian split tests and numerical simulation by discrete element method for heterogeneous gneiss with bedding structure. Chin J Mech Eng.

[CR65] Tomasone P, Bahrani N, Hadjigeorgiou J (2020) Practical considerations in the modelling of resin-grouted rockbolts. J S Afr Inst Mining Metall 120(6). 10.17159/2411-9717/1010/2020

[CR66] Varden R, Lachenicht R, Player J, Thompson A, Villaescusa E(2008) Development and implementation of the Garford dynamic bolt at the Kanowna Belle Mine. In: Proceedings of the 10th Underground operators conference

[CR67] Wang H, Jiang Y, Xue S, Shen B, Wang C, Lv J, Yang T (2015). Assessment of excavation damaged zone around roadways under dynamic pressure induced by an active mining process. Int J Rock Mech Min Sci.

[CR68] Wang J, Apel DB, Pu Y, Hall R, Wei C, Sepehri M (2021). Numerical modeling for rockbursts: A state-of-the-art review. J Rock Mech Geotech.

[CR69] Wang J, Apel DB, Dyczko A, Walentek A, Prusek S, Xu H, Wei C (2021). Investigation of the rockburst mechanism of driving roadways in close-distance coal seam mining using numerical modeling method. Min Metall Explor.

[CR70] Wang Q, Jiang B, Pan R, Li SC, He MC, Sun HB, Qin Q, Yu HC, Luan YC (2018). Failure mechanism of surrounding rock with high stress and confined concrete support system. Int J Rock Mech Min Sci.

[CR71] Wu R, Oldsen J, Campoli A(2011) Application of yield-lok bolt for bursting and convergence grounds in mines. In: 30th International Conference on Ground Control in Mining. Morgantown, pp 26–28

[CR72] Wu Y, Gao F, Chen J, He J (2019). Experimental study on the performance of rock bolts in coal burst-prone mines. Rock Mech Rock Eng.

[CR73] Yang SQ, Chen M, Jing HW, Chen KF, Meng B (2017). A case study on large deformation failure mechanism of deep soft rock roadway in Xin’An coal mine, China. Eng Geol.

[CR74] Yokota Y, Zhao Z, Nie W, Date K, Iwano K, Koizumi Y, Okada Y(2019) Development of a new deformation-controlled rockbolt: numerical modelling and laboratory verification. In: Proceedings of the Ninth International Symposium on Ground Support in Mining and Underground Construction. Australian Centre for Geomechanics, Perth, Australia, pp 545–556

[CR75] Zhang C, Feng XT, Zhou H, Qiu S, Wu W (2012). Case histories of four extremely intense rockbursts in deep tunnels. Rock Mech Rock Eng.

[CR76] Zhang P, Nordlund E(2019) Numerical investigation of dynamic response of a rockbolt under drop testing and simulated seismic loading conditions. In: Proceedings of the Ninth International Symposium on Ground Support in Mining and Underground Construction. Australian Centre for Geomechanics, Perth, Australia, pp 387–398

[CR77] Zhao T, Xing M, Guo W, Wang C, Wang B (2021). Anchoring effect and energy-absorbing support mechanism of large deformation bolt. J Cent South Univ.

[CR78] Zhu D, Wu Y, Liu Z, Dong X, Yu J (2020) Failure mechanism and safety control strategy for laminated roof of wide-span roadway. Eng Fail Anal 111. 10.1016/j.engfailanal.2020.104489

